# HDAC6 and CXCL13 Mediate Atopic Dermatitis by Regulating Cellular Interactions and Expression Levels of miR-9 and SIRT1

**DOI:** 10.3389/fphar.2021.691279

**Published:** 2021-09-13

**Authors:** Yoojung Kwon, Yunji Choi, Misun Kim, Myeong Seon Jeong, Hyun Suk Jung, Dooil Jeoung

**Affiliations:** ^1^ Department of Biochemistry, Kangwon National University, Chuncheon, Korea; ^2^ Chuncheon Center, Korea Basic Science Institute, Chuncheon, Korea

**Keywords:** atopic dermatitis, cellular interactions, HDAC6, CXCL13, SIRT1, miR-9

## Abstract

Histone deacetylase 6 (HDAC6) has been known to regulate inflammatory diseases. The role of HDAC6 in allergic skin inflammation has not been studied. We studied the role of HDAC6 in atopic dermatitis (AD) and the mechanisms associated with it. The decreased expression or chemical inhibition of HDAC6 suppressed AD by decreasing autophagic flux and cellular features of AD. AD increased expression levels of the Th1 and Th2 cytokines, but decreased expression levels of forkhead box P3 (FoxP3) and interleukin-10 (IL-10) in an HDAC6-dependent manner. CXC chemokine ligand 13 (CXCL13), which was increased in an HDAC6-depenednt manner, mediated AD. MiR-9, negatively regulated by HDAC6, suppressed AD by directly regulating the expression of sirtuin 1 (SIRT1). The downregulation or inhibition of SIRT1 suppressed AD. Experiments employing culture medium and transwell suggested that cellular interactions involving mast cells, keratinocytes, and dermal fibroblast cells could promote AD; HDAC6 and CXCL13 were found to be necessary for these cellular interactions. Mouse recombinant CXCL13 protein increased HDAC6 expression in skin mast cells and dermal fibroblast cells. CXCL13 protein was found to be present in the exosomes of DNCB-treated skin mast cells. Exosomes of DNCB-treated skin mast cells enhanced invasion potentials of keratinocytes and dermal fibroblast cells and increased expression levels of HDAC6, SIRT1 and CXCL13 in keratinocytes and dermal fibroblast cells. These results indicate that HDAC6 and CXCL13 may serve as targets for the developing anti-atopic drugs.

## Introduction

Atopic dermatitis (AD) is a chronic, recurrent, non-infectious disease characterized by persistent itching of the skin. AD is thought to be a heterogeneous disease driven by both genetic and environmental factors. AD is characterized by epidermal barrier dysfunction and IgE-mediated sensitization to allergens. Epidermal barrier dysfunction includes reduced water retention (Flohr et al., 2010) and increased susceptibility to infections (Miajlovic et al., 2010). Filaggrin mutations contribute to the increased skin permeability seen in patients with AD (Irvine et al., 2011). Epidermal barrier dysfunction induces keratinocytes to release cytokines such as IL-1β and thymic stromal lymphopoietin (TSLP) (Hou et al., 2021). TSLP stimulates the production of TH2 cytokines (IL-4, IL-5, and IL-13) in naïve T cells (Kabata et al., 2020). Epidermal barrier dysfunction also results in inflammation by activating inflammatory dendritic cells and initiating TH2 cell-mediated response (Sun et al., 2021). Activated TH2 cells releases TH2 cytokines, which promote IgE class switching (Akdis et al., 2020). The acute phase of AD is characterized by infiltration of skin-homing of TH2 cells. TH1 cells are thought to play role in chronic phase of AD (Cole et al., 2014).

Mast cells are the key effector cells in IgE-mediated immediate hypersensitivity and allergic diseases (Kim et al., 2021). AD skin lesions contain infiltrating CD4^+^ T cells, mast cells, eosinophils, Langerhans cells, and inflammatory dendritic epidermal cells (IDEC) (Herrmann et al., 2021; Nunomura et al., 2021). The increased serum IgE concentration is correlated with the development of AD skin lesion (Matsuda et al., 1997). FcεRI bound to IgE antibody induces mast cells to secrete chemicals, lipid mediators, and various TH1/TH2 cytokines (Park et al., 2021; Wang et al., 2021). In mouse model of AD employing ovalbumin, mast cell-derived IL-13 inhibits the TH1 response (Leyva-Castillo et al., 2021). In Nc/Nga mouse model of AD, the severity of AD is correlated with the suppression of Treg cells (Lee et al., 2021).

In addition to gene mutations, epigenetic modifications contribute to the pathogenesis of AD (Yi and Mcgee, 2021). Epigenetic modifications include DNA methylations, histone modifications, and microRNAs. Epigenome differences between AD patients and healthy people have been reported (Mu and Zhang, 2020). In AD, epigenetic modifications target genes involved in immune regulation, genes of innate immunity, and genes encoding epidermal structural proteins (Sroka-Tomaszewska et al., 2021).

Histone modifications play important roles in various inflammatory diseases, including asthma and contact hypersensitivity (Ran and Zhou, 2019). Histone modifications contribute to the pathogenesis of allergic diseases by regulating T cells, macrophages, and fibroblasts (Alaskhar Alhamwe et al., 2018). Trichostatin A, an inhibitor of histone deacetylases (HDACs), suppresses the induction of AD by decreasing IL-4 level and increasing Treg population (Kim et al., 2010).

HDAC6 primarily deacetylates non-histone proteins, including α-tubulin, cortactin, heat shock protein 90 (HSP90), and peroxiredoxin (Prx1) (Vergani et al., 2019; Leyk et al., 2017). Histone deacetylase 6 (HDAC6) plays critical role in inflammatory diseases (Ren et al., 2016; Sellmer et al., 2018) by regulating the levels of inflammatory cytokines.

Tubastatin A (TubA), a selective HDAC6 inhibitor, alleviates airway inflammation by decreasing expression of interleukin-4 (IL-4) and interleukin-5 (IL-5) (Ren et al., 2016). HDAC6 activates cyclooxygenase-2 (COX-2)/prostaglandin E2 (PGE2) signaling pathway (Zhang J. et al., 2019). COX-2 mediates anaphylaxis by regulating miR-26a/-26b (Kwon et al., 2015). HDAC6 increases the expression of Beclin1, a mediator of autophagy, by activating c-Jun N-terminal kinases (JNK) (Jung et al., 2012). P62, a selective receptor of autophagy, binds to HDAC6 and regulates acetylation of alpha tubulin and cortactin (Yan et al., 2013). P62 mediates cellular interactions during anaphylaxis (Kim et al., 2019). Anaphylaxis shares common molecular features with AD (Kim et al., 2018) and herein we suggest a role for HDAC6 in AD.

Sirtuin-1 (SIRT1) belongs to the type III histone deacetylases. SIRT1/nuclear factor-κB (NF-κB) signaling is necessary for allergic rhinitis (Niu et al., 2020). SIRT1 contributes to the pathogenesis of asthma by promoting autophagy (Wu et al., 2020). While an increased expression of SIRT1 has been reported in a murine model of AD (Lee et al., 2020), the role of SIRT1 in AD have not been studied in sufficient detail.

In this study, we showed that HDAC6 mediated AD by regulating the expression levels of CXCL13, miR-9, and SIRT1. CXCL13 and SIRT1 were shown to mediate AD. miR-9 targeted SIRT1 and was shown to suppress AD. Experiments employing culture medium and transwell showed that cellular interactions involving mast cells, and fibroblast cells would mediate AD. Roles of HDAC6 and CXCL13 in mediating these cellular interactions were investigated. We also showed the role of CXCL13-containing exosomes in mediating cellular interactions.

## Materials and Methods

### Materials

Chemicals used in this study were purchased from Sigma-Aldrich (United States). We purchased anti-mouse and anti-rabbit IgG horseradish peroxidase (HRP)-conjugated antibodies from Thermo Pierce (Rockford, IL, United States). We purchased siRNAs and primers from Bioneer Company (South Korea). The mouse miR-9 mimic was purchased from Dharmacon Inc. (United States). Mouse recombinant CXCL13 protein was purchased from R&D Systems.

### Cell Culture

HaCaT cells were purchased (HDFa lot #1780051, Gibco, United States) and expanded in Dulbecco’s modified eagle medium (DMEM; Gibco) containing 8% fetal bovine serum. Skin mast cells (Kim et al., 2018) and dermal fibroblast cells (Seluanov et al., 2010) were isolated according to the standard procedures. For isolation of mouse skin dermal fibroblast cells, the underarm area of Nc/Nga mouse was shaved and cut off and the tissue fragments were transferred to culture dish. Tissue fragments were cut into 1 mm pieces and incubated for 1 h at 37°C with liberase TM solution (Sigma). After digestion, DMEM/F12 medium was added to stop liberase digestion. Centrifugation to remove liberase was followed and the pellet was resuspended in DMEM/F12 media. After washing, the final pellet was resuspended in media and incubation was continued on cell plates for 7 days. Fibroblasts start to exit from tissue fragment within 5 days. Dermal fibroblasts at passage four or five were used in this study.

### Animals

We purchased female Nc/Nga mice from Nara Biotech (South Korea). and Mice were group housed under specific pathogen-free conditions. Animal experiments performed in this study were approved by the Institutional Animal Care and Use Committee (IACUC) of the Kangwon National University.

### Induction of Atopic Dermatitis

NC/Nga mice were randomly divided into three groups (*n* = 5 per group) and the upper backs of mice were shaved with a clipper (day −1). During days 0–7, 150 µl of 1% DNCB (2, 4-dinitrochloro benzene) solution was applied topically once in 3 days. Later, the same volume of 0.5% or 1% DNCB was applied three times a week. Symptoms of AD were induced by using DNCB. Dermatitis scores of 0 (none), 1 (mild), 2 (moderate), and 3 (severe) were given for each of the four symptoms: dryness, excoriation, erosion, and erythema and edema. The sum of the individual scores was used as the clinical severity. To investigate the effect of HDAC6, CXCL13 or miR-9 on AD, the Nc/Nga mice were injected intravenously with siRNA or miRNA mimics (1 μM) in a total of five times as described in the timeline of each experiment. To investigate the effect of tubastatin A or sirtinol on AD, the mice were injected intraperitoneally with tubastatin A (25 mg/kg) or sirtinol (0.5 mg/kg) in a total of five times as described in the timeline of each experiment.

### Induction of AD by Oxazolone

Female SKH-1 mice, aged 6–8 weeks, were sensitized with topical applications with 50 μl 2% Oxazolone (Ox). After 1 week, mice were treated topically with 50 μl 0.25% Ox three times a week for an additional 7 weeks (total of 21 exposures).

### The Levels of PGE2, Histamine Released, and CXCL13

The level of PGE2 was measured according to the manufacturer’s instruction using ELISA kit (Abcam). Histamine release assays were performed according to the manufacturer’s instruction (Enzo). The level of CXCL13 was determined according to the manufacturer’s instruction (R&D System).

### MicroRNA Array

The miRNA Array III (Signosis, CA, United States) was used for miRNA expression analysis. Total miRNA was hybridized to 132 miRNA oligonucleotide probes. The level of miRNA was determined by Streptavidin-HRP chemiluminescence.

### MiRNA Extraction and Quantitative Real-Time PCR

Total miRNA was isolated with the miRNeasy Micro Kit (Qiagen, CA, United States). The Reverse transcription of the extracted miRNA was performed using a miScript II RT Kit (Qiagen) with universal RT primer. The expression level of miR-9 was quantified with SYBR Green Master Mix (Qiagen). Expression level of miR-9 was defined based on the threshold (Ct), and relative expression levels were calculated as 2^−^(^Ct^ofmiR^−9^)^−^(CtofU^6^) after normalization with reference to expression of U6 small nuclear RNA. Primer sequences are provided in Supplemental Table S1.

### Cytokine Array

Identification of HDAC6-regualted cytokines was by using a Proteom ProfilerTM Mouse Cytokine Array Kit (R and D Systems).

### Transfection

The transfection was performed using the jetPRIME transfection reagent (Polyplus, United States). The mouse HDAC6 siRNA [5′- CUG​AGU​ACG​UGG​AGC​AUC​U -3′ (sense) and 5′-AGA​UGC​UCC​ACG​UAC​UCA​G -3′ (antisense)]; mouse CXCL13 siRNA [5′-GUG​ACA​ACC​CAC​UUC​AGA​U -3′ (sense) and 5′- AUC​UGA​AGU​GGG​UUG​UCA​C-3′ (antisense)]; mouse SIRT1 siRNA [5′-CAC​AAG​ACU​CUU​CUG​UGA​U -3′ (sense) and 5′-AUC​ACA​GAA​GAG​UCU​UGU​G -3′ (antisense)]; Human SIRT1 siRNA [5′-GAC​UCU​GAA​GAU​GAC​GUC -3′ (sense) and 5′-AGA​CGU​CAU​CUU​CAG​AGU​C-3′ (antisense)] were used. The negative control siRNA was purchased from Bioneer Company (cat.SN-1002). For *in vivo* transfections, in vivo-jetPEI^®^ (Polyplus, cat.201-10G) was used. The sequences of miR-9 mimic (Dharmacon) are [5′- UCU​UUG​GUU​AUC​UAG​CUG​UAU​GA-3′.

### Luciferase Activity Assays

PCR-amplified SIRT1 gene segment encompassing 3′-UTR was cloned into the XbaI site of pGL3 luciferase plasmid. The mutant pGL3-3′-UTR-SIRT1 construct was made with the QuikChange site-directed mutagenesis kit (Stratagene). Luciferase activity assay was performed according to the standard procedures (Kim et al., 2019).

### Reactive Oxygen Species Measurement

Cells were treated with or without tubastatin A (10 μM) for 1 h, DNCB (5 μM) was then added to the cells, and incubation was continued for 1 h. Subsequently, DCFH-DA solution (10 μM) was added to each well. Forty minutes later, the fluorescence level of the 2′, 7′-dichlorofluorescein (DCF) product was quantified by fluorescence microscope.

### Chromatin Immunoprecipitation

Assays were performed according to the manufacturer’s instruction (Upstate). PCR was done on the phenol/chloroform-extracted DNA with specific primers of the SIRT1 promoter-1 (5′- GGG​GTA​GTA​AAA​GCT​GGC​CA -3′ (sense) and 5′- ATT​GTC​TGC​CTT​CAT​AGC​TAG​TGC -3′ (antisense)), SIRT1 promoter-2 (5′- GCA​CTA​GCT​ATG​AAG​GCA​GAC​AAT-3′ (sense) and 5′-GAC​TAT​ACA​TGG​ACT​GAC​CCT​GG -3′ (antisense)), and SIRT1 promoter-3 (5′-GGT​AGT​GGC​TTA​GTC​CCT​GG -3′ (sense) and 5′-CAC​CCC​ATT​AAA​TTG​TCT​GCC​TTC -3′ (antisense)) sequences were used to examine the binding of NF-kB to the SIRT1 promoter sequences.

### Immunofluorescence Staining

Cells were incubated with primary antibody specific to LC3 (1:100; Santa Cruz Biotechnology) for 2 h. Anti-rabbit Alexa Fluor 488 (for detection of LC3) secondary antibody (Molecular Probes) was added to cells and incubated for 1 h. Fluorescence images were acquired using a confocal laser scanning microscope and software (Fluoview version 2.0) with a ×60 objective (Olympus FV300, Japan).

### Immunoblot and Immunoprecipitation

Immunoblot and immunoprecipitation were performed according to the standard procedures (Kim et al., 2018). The following primary antibodies were used: anti-HDAC6, TLR2, TLR4, and SIRT1 (ABclonal); anti-FcεRIβ, Lyn, GATA3, T-bet, JNK1, pJNK1^T183/Y185^, Tryptase, Chymase, BECN1, MyD88, TSG101, and Calnexin (Santa Cruz Biotechnology); anti-CXCL13 (R&D Systems); anti-CD163, FoxP3, TSLP, and MIP-2 (Abcam); anti-iNOS, pBECN1^S14^, COX2, ERK1/2, pERK^T204^, HDAC3, NFκB, AMPKα, pAMPKα^T172^, IKBα, pIKBα^S32^, p38MAPK, p-p38MAPK^T180/Y182^, and LC3(Cell Signaling Technology). The detailed information of primary antibodies is described in Supplemental Table S2.

To isolate tissue lysates, tissue was frozen in liquid nitrogen to preserve protein structure and homogenized with RIPA buffer. After lysis, vortexing and centrifugation at 10,000 X g for 15 min at 4°C were followed. Supernatant was then obtained and used as tissue lysates for immunoblot and immunoprecipitation.

### Invasion

Invasion was determined by using a transwell chamber system (Corning, NY, United states). Trypsinized cells (1x10^4^) in DMEM with 0.1% bovine serum albumin were added to each upper chamber of the transwell (Corning). Culture medium of skin mast cells, HaCaT, or dermal fibroblast cells was placed in the lower chamber, and cells were incubated at 37°C for 12 h. The invaded cells were stained and counted.

### Histological Examination

Skin tissues were fixed with 10% (vol./vol.) buffered formalin, and embedded in paraffin. Sections (5 μm thickness) were stained with hematoxylin and eosin (H and E) or toluidine blue for leukocyte infiltration or mast cell degranulation, respectively.

### Immunohistochemical Staining

Immunohistochemical staining of tissues was performed using an avidin-biotin detection method (Vectastain ABC kit, Vector Laboratories Inc. Burlingame, CA, United States). The following primary antibodies were used: anti-CD163 (1:100, Abcam); anti-iNOS (1:100, Cell signaling); anti-CXCL13 (1:250, R and D Systems); anti-HDAC6 (1: 300, Abcam); anti-chymase (1:100, Santa Cruz Biotechnology); anti-SIRT1 (1:100, Santa Cruz Biotechnology); anti-tryptase (1:100, Santa Cruz Biotechnology), and anti-pBeclin1^S14^ (1:100, Cell signaling). After washing, biotinylated secondary antibodies were added (1:100 or 1:200 dilutions) for 1 h. Diaminobenzidine (Vector Laboratories, Inc.) was used for color development. Sections were counterstained with Mayer’s hematoxylin. The detailed information of primary antibodies is described in Supplemental Table S2.

### Electron Microscopic Observation of Autophagosomes

Skin mast cells of BALB/C mouse were treated with or without DNCB (5 μM) for 1 h. Cells were then mixed with fixing solution (2.5% glutaraldehyde in 0.1 M cacodylate solution (pH 7.0)) for 1 h, and then treated with 2% osmium tetroxide for 2 h at 4°C. Cells were dehydrated with a graded acetone series, embedded into Spurr medium (Electron Microscopy System), and were sectioned (60 nm) with an ultra-microtome (RMC MTXL, Arizona, United States), and double-stained with 2% uranyl acetate for 20 min and lead citrate for 10 min. The sections were then viewed under a Tecnai G2 (FEI, United States) TEM at 200 kV.

### Isolation and Characterization of Exosomes

The culture medium of skin mast cells, treated with or without DNCB (5 μM) for 24 h, was harvested, and the exosomes were purified using Total Exosomes Isolation Reagent from cell culture media (Invitrogen). Exosomes were viewed under a Tecnai T10 transmission electron microscope (FEI, United States). The size of the exosomes was measured with a nanoparticle tracking analysis (NTA) (NANOSIGHT, United Kingdom).

### Internalization of Exosomes

Exosomes isolated from skin mast cells treated with or without DNCB (5 μM) for 24 h were labeled using PKH67 Fluorescent Cell Linker kits (Sigma-Aldrich). PKH67-labeled exosomes (2 μg) or unlabeled exosomes (2 μg) were added to HaCaT and dermal fibroblast cells. Twenty-four hours later, cells were fixed with 4% paraformaldehyde solution and visualized under EVOS FL Auto 2 (ThermoFisher).

### The Presence of CXCL13 in the Exosomes of DNCB-Treated RBL2H3 Cells

Immuno-EM was performed to examine the presence of CXCL13 in the exosomes. Primary rabbit or/and mouse antibodies (Anti-CXCL13 or/and Anti-TSG1 antibodies) at 1:20 dilutions were used. The grid was incubated in secondary antibodies, anti- Rabbit IgG conjugated to 10 nm and anti-mouse IgG conjugated to 25 nm (AURION, Holland) diluted 1:20 in 0.1% BSA-PBS. The sample grids were stained with uranyl acetate and lead citrate. The sectioned and immune-gold labeled grids were examined using a Tecnai T10 transmission electron microscope (FEI, United States) operated at 100 kV and JEOL-2100 F transmission electron microscope (JEOL, United States) operated at 200 KV.

### Image Analysis

Analysis of integral optical density (IOD) was performed using ImageJ. The DAB color channel was deconvoluted from the hematoxylin color channel using the “IHC Toolbox” plug-in. the DAB image was segmented by thresholding and the software analyzes the gray level (GL) of every pixel of the digitized image. Optical density (OD) can be calculated from the GL through the relation OD = –log (GL (object)/GL (max)), where GL (max) is the mean GL of a reference region of maximal transmittance. The IOD of a labeled section is calculated multiplying the pixel mean gray value of the area by the area of the region of the object. IOD represents a value that takes into account both the intensity of the DAB staining and the labeled areas.

### Statistical Analysis

Data analysis was performed using the GraphPad Prism statistics program (Version 7, GraphPad Prism software). Results were presented as means and standard error values (SE). Student’s t tests were performed for comparisons between two groups. One-way ANOVA was carried out for comparisons among three or more groups and was followed by Tukey’s post hoc test. Statistical significance was defined as *p* value less than 0.05.

## Results

### HDAC6, Induced by AD, Mediates AD

**FIGURE 1:**
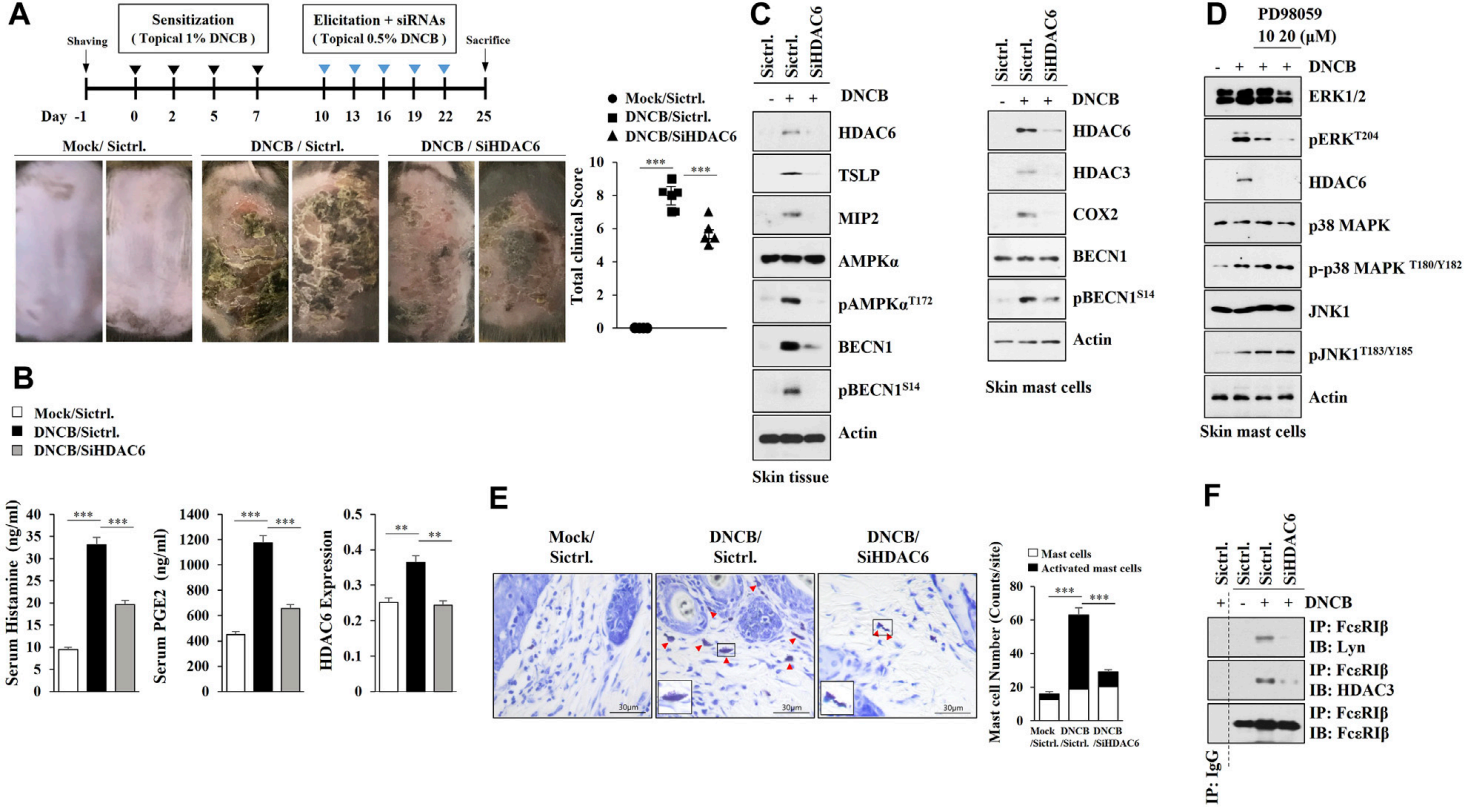
HDAC6 mediates AD. **(A)** Shows experimental timeline to determine the effect of HDAC6 on AD (upper). AD was induced as described. Nc/Nga mice were given an intravenous injection of HDAC6 siRNA on the indicated days of the timeline. Each experimental group comprised four Nc/Nga mice. Representative images were shown. SiCtrl. denotes negative control siRNA. ***, *p* < 0.001. **(B)** HDAC6 regulates serum PGE2 level and the amount of histamine released during AD. QRT-PCR employing tissue lysates was also performed. **, *p* < 0.01; ***, *p* < 0.001. Average values of three independent experiments were shown. **(C)** Representative blots of three independent experiments were shown. Immunoblot of skin tissue (left) and skin mast cells isolated from skin tissue of Nc/Nga mouse of each experimental group (right) were performed. **(D)** Skin mast cells isolated from DNCB-untreated Nc/Nga mouse were treated with DNCB (5 μM) in the absence or presence of PD98059 for 1 h. Representative blots of three independent experiments were shown. **(E)** Skin tissues of Nc/Nga mice of each experimental group were subjected to toluidine blue staining. Closed triangle represents degranulated mast cells. Enlarged version of degranulated mast cells was also shown. ***, *p* < 0.001 **(F)** Immunoprecipitation using skin tissue lysates was performed. Immunoprecipitation employing isotype-matched IgG antibody (2 μg/ml) was also performed. Representative blots of three independent experiments were shown.

Several reports have implied the role of HDAC6 in atopic dermatitis (AD) but failed to link this fully, thus, we examined the role of HDAC6 in AD. AD in Nc/Nga mice was induced by 2, 4-dinitrochlorobenzene (DNCB) (Figure 1A), and the downregulation of HDAC6 was shown to attenuate the clinical symptoms associated with AD (Figure 1A). AD increased the amount of histamine released and serum prostaglandin E2 (PGE2) levels in an HDAC6-dependent manner (Figure 1B). AD increased hallmarks of allergic inflammation, such as Macrophage inflammatory protein-2 (MIP-2), Thymic stromal lymphopoietin (TSLP), HDAC3, and COX2, and autophagic flux such as pAMPKα^T172^ and pBeclin1^S14^, in an HDAC6-dependent manner (Figure 1C, left). Immunoblot of skin mast cells isolated from skin tissue of Nc/Nga mouse of each experimental group also showed that AD increased pBeclin1^S14^, in an HDAC6-dependent manner (Figure 1C, right). DNCB increased the expression of HDAC6 via ERK in skin mast cells isolated from DNCB-untreated Nc/Nga mouse (Figure 1D). Toluidine blue staining showed that AD increased the number of degranulated mast cells in an HDAC6-dependent manner (Figure 1E). Immunoprecipitation of skin tissue lysates showed that AD induced the binding of FcεRI to Lyn in an HDAC6-depndent manner (Figure 1F). AD induced epidermal hyperplasia in an HDAC6-dependent manner (Supplementary Figure S1A). Immunohistochemical staining showed that AD increased the expression levels of CD163 and chymase, but decreased the expression of inducible nitric oxide synthase (iNOS), in an HDAC6-dependent manner (Supplementary Figure S1A). CD163 and iNOS are markers of M2 and M1 macrophages, respectively. Immunoblot of skin tissue lysates showed that AD increased the expressions of T-bet and GATA3, but decreased Forkhead box P3 (FoxP3) expression in an HDAC6-dependent manner (Supplementary Figure S1B). T-bet and GATA-3 are transcriptional factors of Th1 and Th2 cells, respectively. FoxP3 is a specific transcriptional factor in Treg cells. QRT-PCR analysis of skin tissue lysates showed that AD increased the expression levels of Th1 cytokines (IL-1β, IFN-γ, and TNF-α) and Th2 cytokines (IL-4, IL-5, IL-6, and IL-13), while decreasing the expression of IL-10, an immune suppressive cytokine, in an HDAC6-dependent manner (Supplementary Figure S1C). DNCB enhanced the formation of autophagosomes and autolysosomes in skin mast cells isolated from DNCB-untreated Nc/Nga mouse (Supplementary Figure S1D).

**FIGURE 2:**
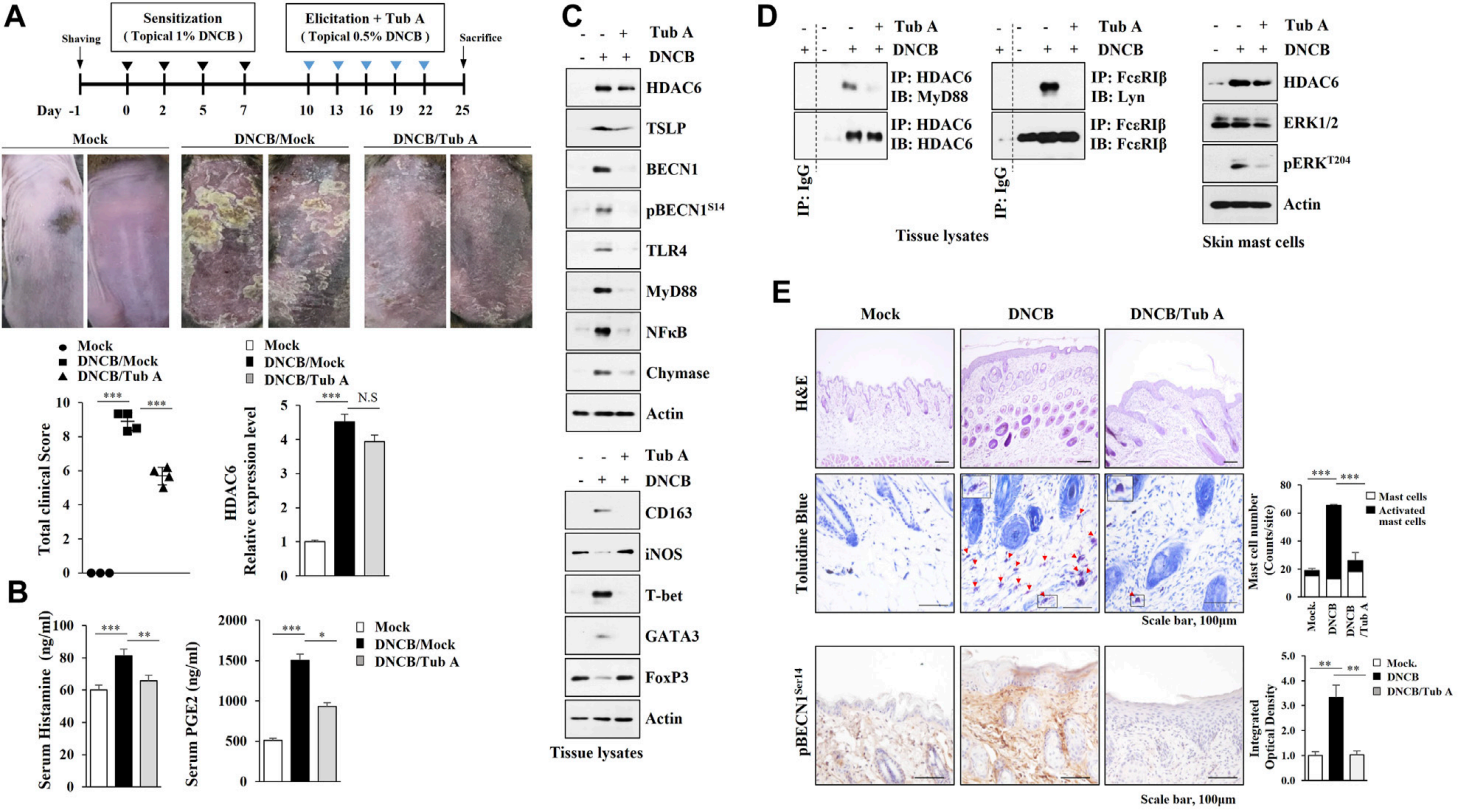
The inhibition of HDAC6 suppresses AD. **(A)** Tubastain A, an inhibitor of HDAC6, attenuates clinical symptoms associated with AD. Nc/Nga mice were given an intraperitoneal injection of tubastatin A (25 mg/kg). Each experimental group comprised four Nc/Nga mice. Tissue lysates were subjected to qRT-PCR. ***, *p* < 0.001. NS denotes not significant. Average values of three independent experiments were shown. **(B)** Serum PGE2 level and the amount of histamine released were determined *, *p* < 0.05; **, *p* < 0.01; ***, *p* < 0.001. Average values of three independent experiments were shown. **(C)** Skin tissue lysates were subjected to immunoblot and immunoprecipitation. Representative blots of three independent experiments were shown. **(D)** Skin tissue lysates were subjected to immunoprecipitation (left, middle). Immunoprecipitation employing isotype-matched IgG antibody (2 μg/ml) was also performed. Skin mast cells were isolated from NC/Nga mouse of each experimental group and immunoblot was performed (right). Representative blots of three independent experiments were shown. **(E)** HDAC6 regulates cellular features of AD. Closed triangle represents degranulated mast cells. Immunohistochemical staining of skin tissues of Nc/Nga mouse of each experimental group was also performed. **, *p* < 0.01; ***, *p* < 0.001. Quantification was performed using ImageJ (NIH).

We next examined whether the inhibition of HDAC6 would suppress AD. Tubastatin A, an inhibitor of HDAC6, attenuated clinical symptoms (Figure 2A). Tubastain A exerted negative effects on the increased amount of histamine released and serum prostaglandin E2 (PGE2) levels (Figure 2B). Tubastatin A prevented AD from increasing ed expression levels of TSLP, Toll-like receptor 4 (TLR4), Myeloid differentiation 88 (MyD88), and NF-κB (Figure 2C), inducing the binding of HDAC6 to MyD88 (Figure 2D, left) and the binding of FcεRI to Lyn (Figure 2D, left). HDAC6 increases the levels of inflammatory cytokines via Mitogen activated protein kinase (MAPK)/NF-κB/AP1 signaling pathways (Youn et al., 2016). Immunoblot of skin mast cells isolated from skin tissue of DNCB-untreated Nc/Nga mouse showed that DNCB increased extracellular regulated kinase (ERK) phosphorylation in an HDAC6-dependent manner (Figure 2D). Tubastatin A prevented AD from increasing epithelial hyperplasia, the number of activated mast cells, and the expression of pBeclin1^S14^ (Figure 2E). These results confirm the role of HDAC6 in AD.

### CXCL13 Is Regulated by HDAC6 and Mediates AD

**FIGURE 3:**
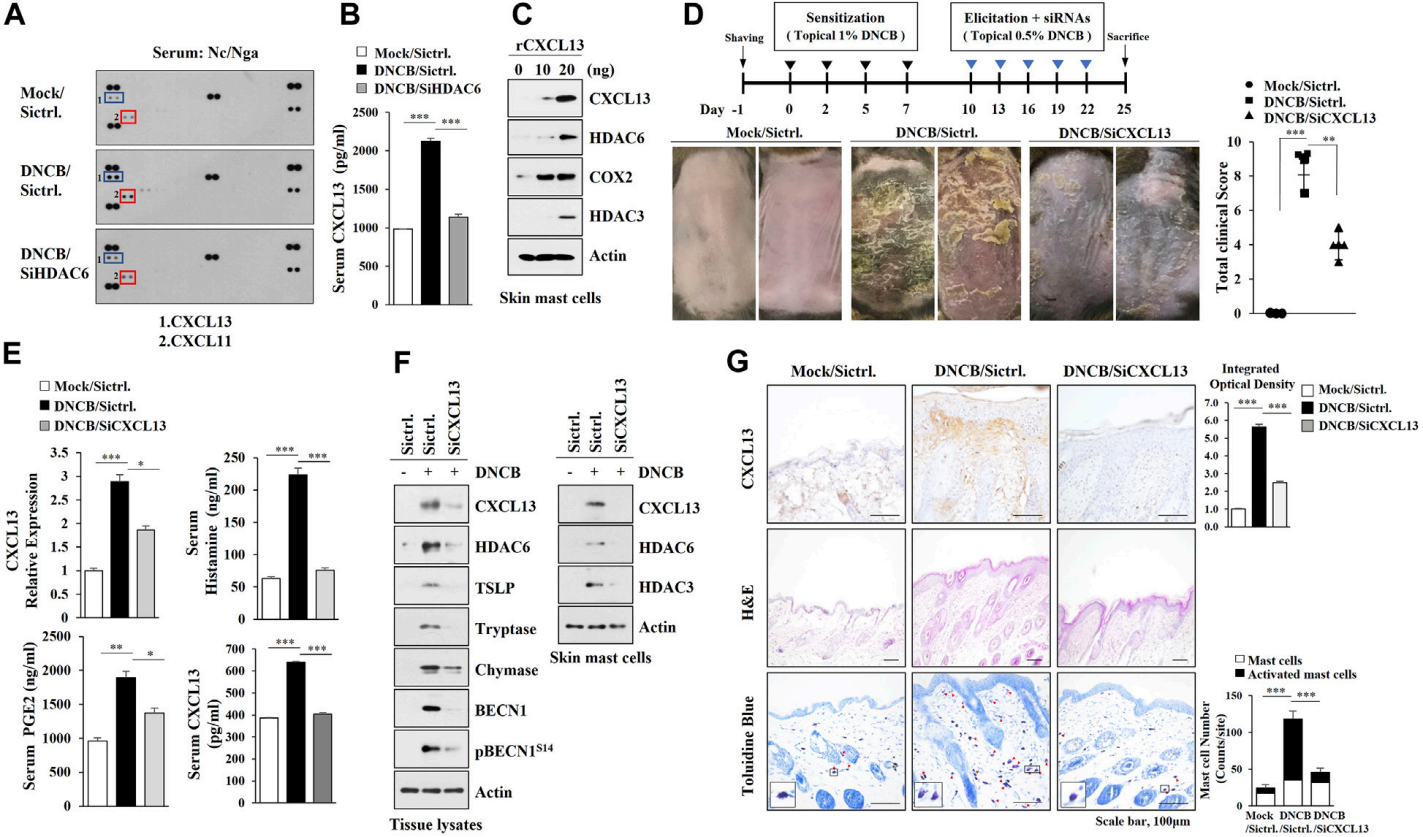
CXCL13, regulated by HDAC6, mediates AD. **(A)** Serum of each Nc/Nga mouse of each experimental group was employed for cytokine array analysis. AD was induced as described. Nc/Nga mice were given an intravenous injection of the indicated siRNA (100 nM). **(B)** ELISA was performed to determine serum CXCL13 level. ***, *p* < 0.001. Average values of three independent experiments were shown. **(C)** Skin mast cells from DNCB-untreated Nc/Nga mouse were treated with CXCL13 as indicated. **(D)** Effect of CXCL13 on clinical symptoms associated with AD was shown. **, *p* < 0.01; ***, *p* < 0.001. **(E)** Serum and tissue lysates were subjected to ELISA and qRT-PCR, respectively. *, *p* < 0.05; **, *p* < 0.01; ***, *p* < 0.001. Average values of three independent experiments were shown. **(F)** Immunoblot analysis employing skin tissue lysates (left) or skin mast cell lysates isolated from skin tissue of Nc/Nga mouse of each experimental group was performed (right). Representative blots of three independent experiments were shown. **(G)** H&E staining, toluidine blue staining, and immunohistochemical staining were performed. Closed rectangle represents degranulated mast cells. ***, *p* < 0.001. Quantification was performed using ImageJ (NIH).

We next wanted to identify HDAC6-regulated cytokines to better understand the mechanisms of AD. AD increased the expression levels of CXCL11 and CXCL13 in an HDAC6-depencdent manner in Nc/Nga mouse model of AD (Figure 3 A, B). The inhibition of HDAC3, a marker of allergic inflammation, suppressesd the expression of IFN-dependent gene such as CXCL11 (Angiolilli et al., 2017). Mouse recombinant CXCL13 protein increased the hallmarks of allergic inflammation in skin mast cells isolated from DNCB-untreated Nc/Nga mouse (Figure 3C) and CXCL13 was necessary for AD (Figures 3D,E). AD increased the molecular features of AD and autophagic flux in an CXCL13-dependent manner (Figure 3F, left). Immunoblot of skin mast cells isolated from skin tissue of Nc/Nga mouse of each experimental group showed that CXCL13 was necessary for the increased HDAC6 expression by AD (Figure 3F, right). CXCL13 was necessary for AD-related cellular features such as increased number of activated mast cells (Figure 3G).

### MiR-9, Negatively Regulated by HDAC6, Suppresses AD

**FIGURE 4:**
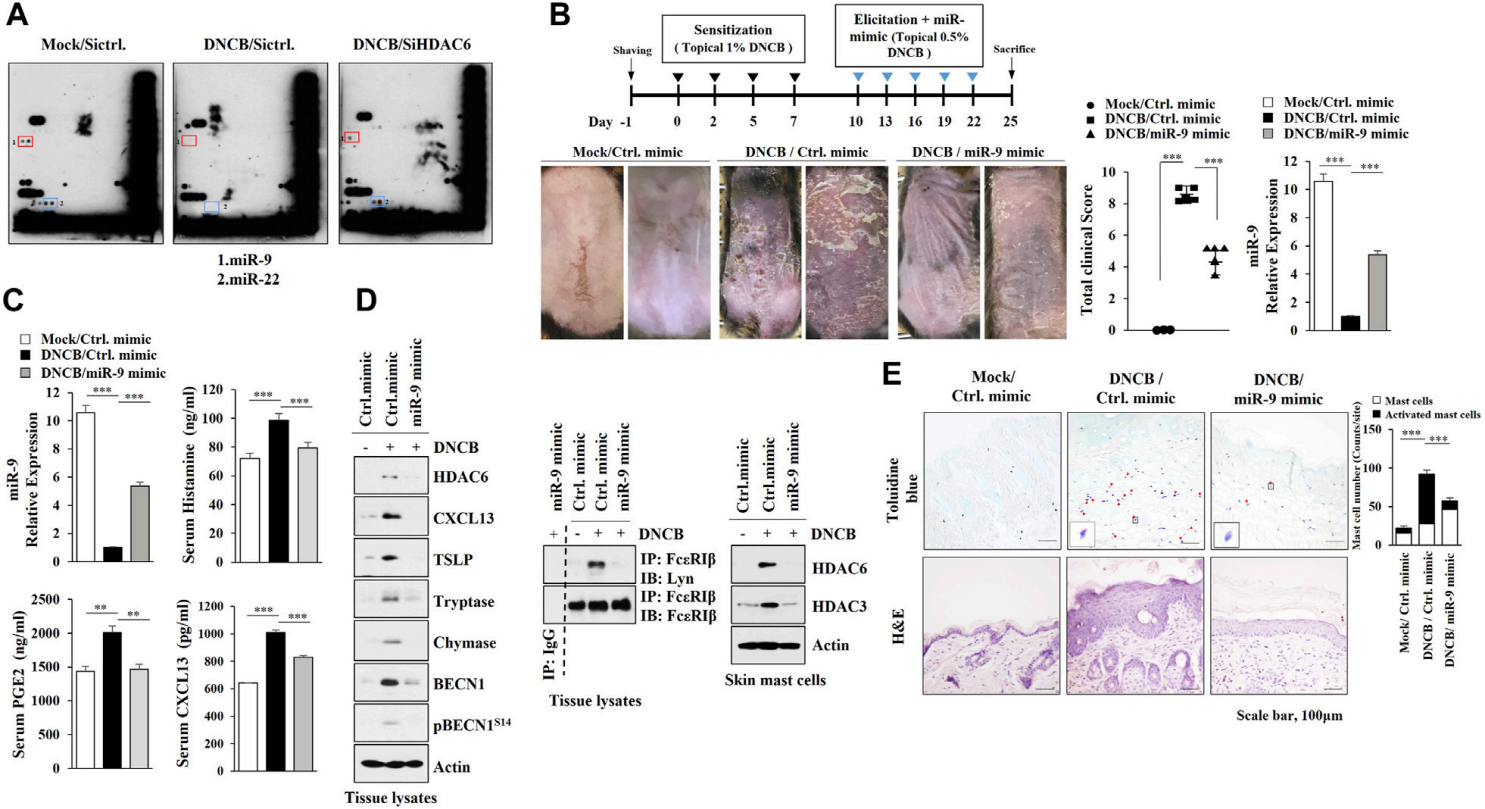
MiR-9, regulated by HDAC6, suppresses AD. **(A)** Skin tissue lysates from each Nc/Nga mouse of each experimental group were subjected to miRNA array analysis. **(B)** MiR-9 mimic suppresses AD. ***, *p* < 0.001. Skin tissue lysates were subjected to qRT-PCR. Average values of three independent experiments were shown. **(C)** ELISA and qRT-PCR were performed. **, *p* < 0.01; ***, *p* < 0.001. Average values of three independent experiments were shown. **(D)** Skin tissue lysates (left) and skin mast cell lysates from skin tissue of Nc/Nga mouse of each experimental group (right) were subjected to immunoblot. Skin tissue lysates were subjected to immunoprecipitation (middle). Immunoprecipitation employing isotype-matched IgG antibody (2 μg/ml) was also performed. Representative blots of three independent experiments were shown. **(E)** H&E staining and toluidine blue staining were performed. Closed triangle represents degranulated mast cells. ***, *p* < 0.001.

Since microRNAs play important roles in AD (Kim et al., 2018), we identified HDAC6-regulated miRNAs. MicroRNA array analysis employing skin tissue lysates of Nc/Nga mouse revealed that AD decreased the expression levels of miR-9 and miR-222 in an HDAC6-dependent manner (Figure 4A). MiR-9 mimic attenuated clinical symptoms associated with AD (Figure 4B) and exerted negative effects on the increased CXCL13 levels, amount of histamine released and serum prostaglandin E2 (Figure 4C). Immunoblot of skin tissue lysates showed that miR-9 mimic inhibited the effect of AD on the molecular features of AD (Figure 4D, left) and the interaction between FcεRI and Lyn (Figure 4D, right). Immunoblot of skin mast cells isolated from skin tissue of Nc/Nga mouse of each experimental group showed that miR-9 mimic inhibited the effect of AD on the increased HDAC6 expression (Figure 4D, right). MiR-9 mimic inhibited cellular features of AD such as increased number of activated mast cells (Figure 4E). These results indicate that miR-9 acts as a negative regulator of AD.

### SIRT1 Acts as a Target of miR-9 and Regulates AD

**FIGURE 5:**
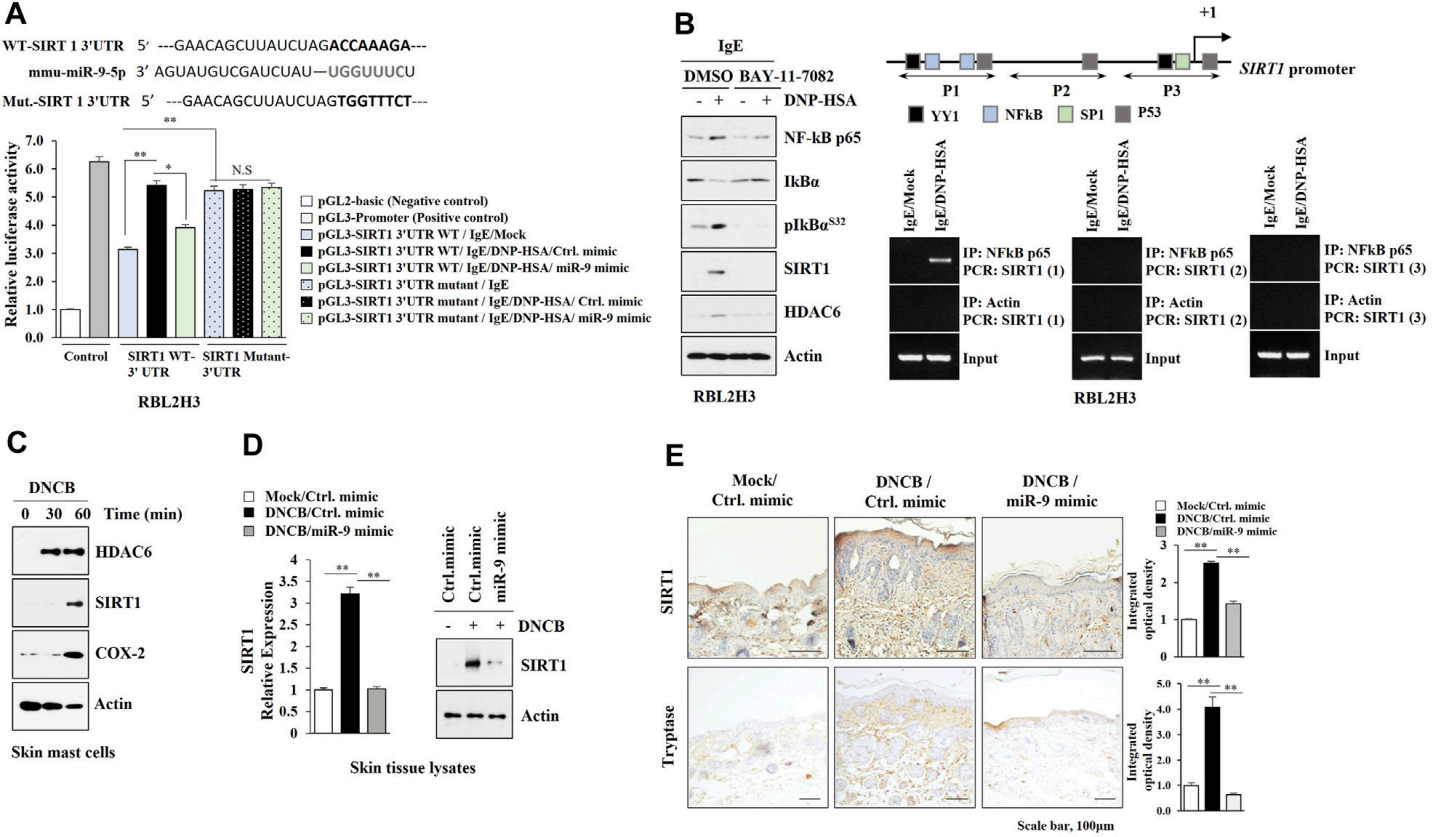
SIRT1 acts as a downstream target of miR-9. **(A)** Wild type Luc-SIRT1 3′-UTR or mutant Luc-SIRT1 3′-UTR was transfected along with the indicated mimic (each at 10 nM) into RBL2H3 cells. The next day, cells were sensitized with DNP-specific IgE (100 ng/ml) for 24 h, and subsequently stimulated with DNP-HSA (100 ng/ml) for 1 h. *, *p* < 0.05; **, *p* < 0.01. NS denotes not significant. Luciferase activity assays were performed. **(B)** IgE-sensitized RBL2H3 cells were pretreated with BAY11-7,082 (10 μM) for 2 h followed by stimulation with DNP-HSA for 1 h (left). IgE-sensitized RBL2H3 cells were stimulated with DNP-HSA for 1 h followed by ChIP assays (right). Potential binding sites of the transcription factors in the promoter sequences of SIRT1 were shown. Numbers in parentheses denote primer binding sites. **(C)** Skin mast cells from DNCB-untreated Nc/Nga mouse were treated with DNCB (5 μM) for various time intervals. **(D)** Skin tissue lysates from each Nc/Nga mouse of each experimental group were subjected to immunoblot and qRT-PCR. **, *p* < 0.01. Representative blots of three independent experiments were shown. **(E)** Immunohistochemical staining was performed. **, *p* < 0.01. Quantification was performed using ImageJ (NIH).

**FIGURE 6:**
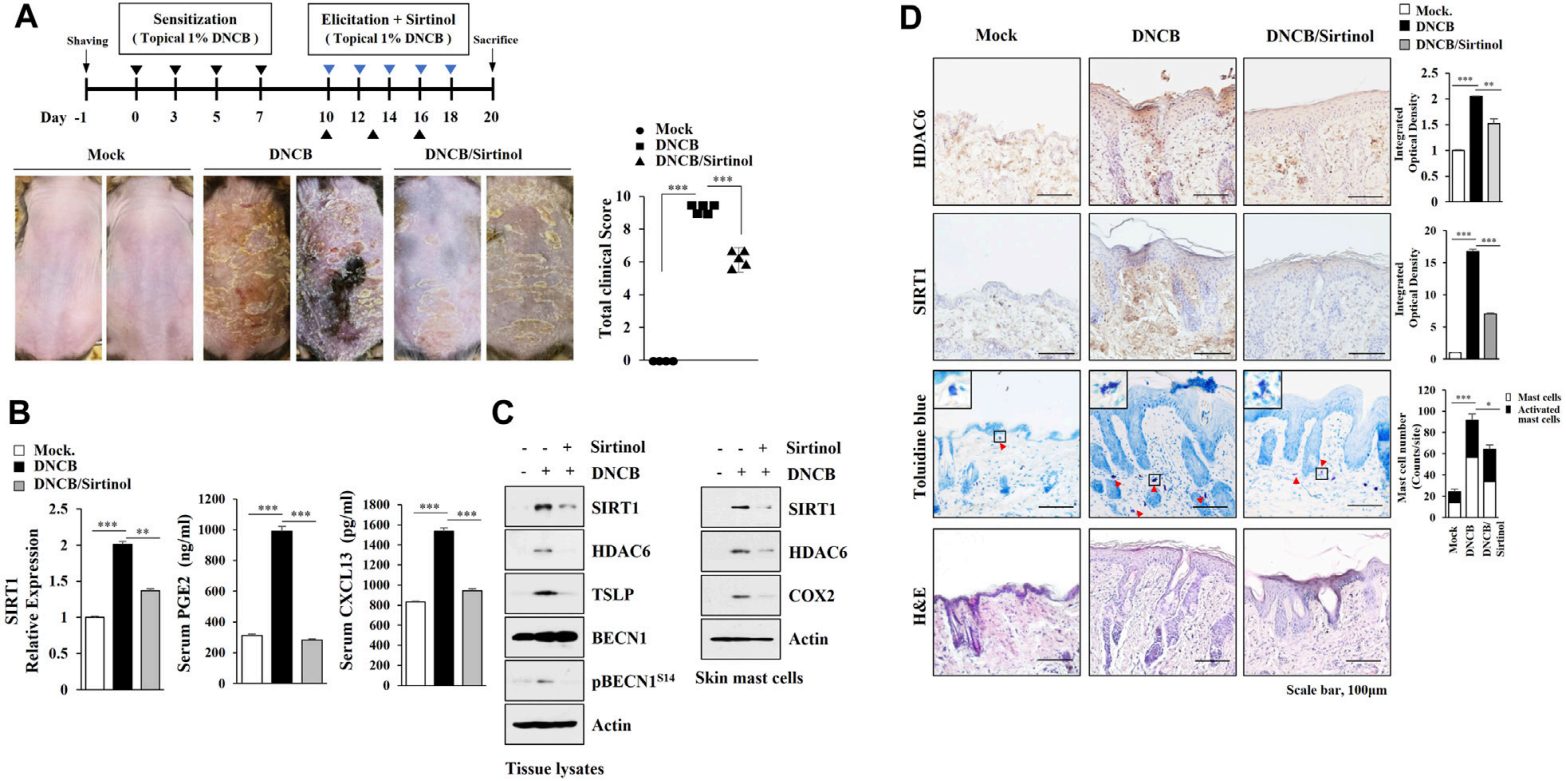
Sirtinol suppresses AD. **(A)** Sirtinol suppresses AD. Nc/Nga mice were given an intraperitoneal injection of sirtinol (0.5 mg/kg). Each experimental group comprised four Nc/Nga mice. ***, *p* < 0.001. Blue triangle denotes intraperitoneal injection with sirtinol. Black triangle denotes topical treatment with DNCB. **(B)** Skin tissue lysates were subjected to qRT-PCR. Serum PGE2 level, and serum CXCL13 level were also determined. **, *p* < 0.01; ***, *p* < 0.001. Average values of three independent experiments were shown. **(C)** Skin tissue lysates were subjected to immunoblot (left). Skin mast cell lysates from Nc/Nga mouse of each experimental group were subjected to immunoblot (right). Representative blots of three independent experiments were shown. **(D)** H&E staining, toluidine blue staining, and immunohistochemical staining were performed. Closed triangle represents degranulated mast cells. *, *p* < 0.05; **, *p* < 0.01; ***, *p* < 0.001. Quantification was performed using ImageJ (NIH).

TargetScan analysis predicted SIRT1 as a target of miR-9. MiR-9 mimic decreased luciferase activity of the wild type SIRT1 3′-UTR, but not luciferase activity of the mutant SIRT1 3′-UTR (Figure 5A). BAY-11-7,082, an NF-kB inhibitor, exerted negative effects on the increased expressions of HDAC6 and SIRT1 in antigen-stimulated RBL2H3 cells (Figure 5B). ChIP assays showed NF-kB binding to the promoter sequences of SIRT1 (Figure 5B). DNCB increased the expression levels of HDAC6 and SIRT1 in skin mast cells isolated from DNCB-untreated Nc/Nga mouse (Figure 5C). Immunoblot and qRT-PCR of skin tissue lysates showed that miR-9 mimic inhibited the effect of AD on the increased SIRT1 expression (Figure 5D). Immunohistochemical staining showed that miR-9 mimic inhibited the effect of AD on the increased expression levels of SIRT1 and tryptase, a marker of allergic inflammation (Figure 5E). Sirtinol, an SIRT1 inhibitor, attenuated clinical symptoms associated with AD (Figure 6A). Sirtinol inhibited the effect of AD on the increased SIRT1 expression, serum PGE2 level and CXCL13 level (Figure 6B). Immunoblot of skin tissue (Figure 6C, left) and skin mast cells isolated from skin tissue of Nc/Nga mouse of each experimental group (Figure 6C, right) showed that sirtinol inhibited the effect of AD on the increased expression levels of SIRT1, HDAC6, TSLP, pBeclin1^S14^, and COX2. Sirtinol inhibited the molecular and cellular features of AD (Figure 6D). We examined whether HDAC6 expression would be increased in another animal model of AD. Oxazolone-induced AD in SKH-1 mice (Supplementary Figure S2A, B) was accompanied by epidermal hyperplasia (Supplementary Figure S2C), an increased number of degranulated mast cells (Supplementary Figure S2C), and the increased expression levels of SIRT1, HDAC6, TSLP, and CXCL13 (Supplementary Figure S2D).

### HDAC6 Regulates the Expression of SIRT1 and Autophagic Flux in Human Keratinocytes

**FIGURE 7:**
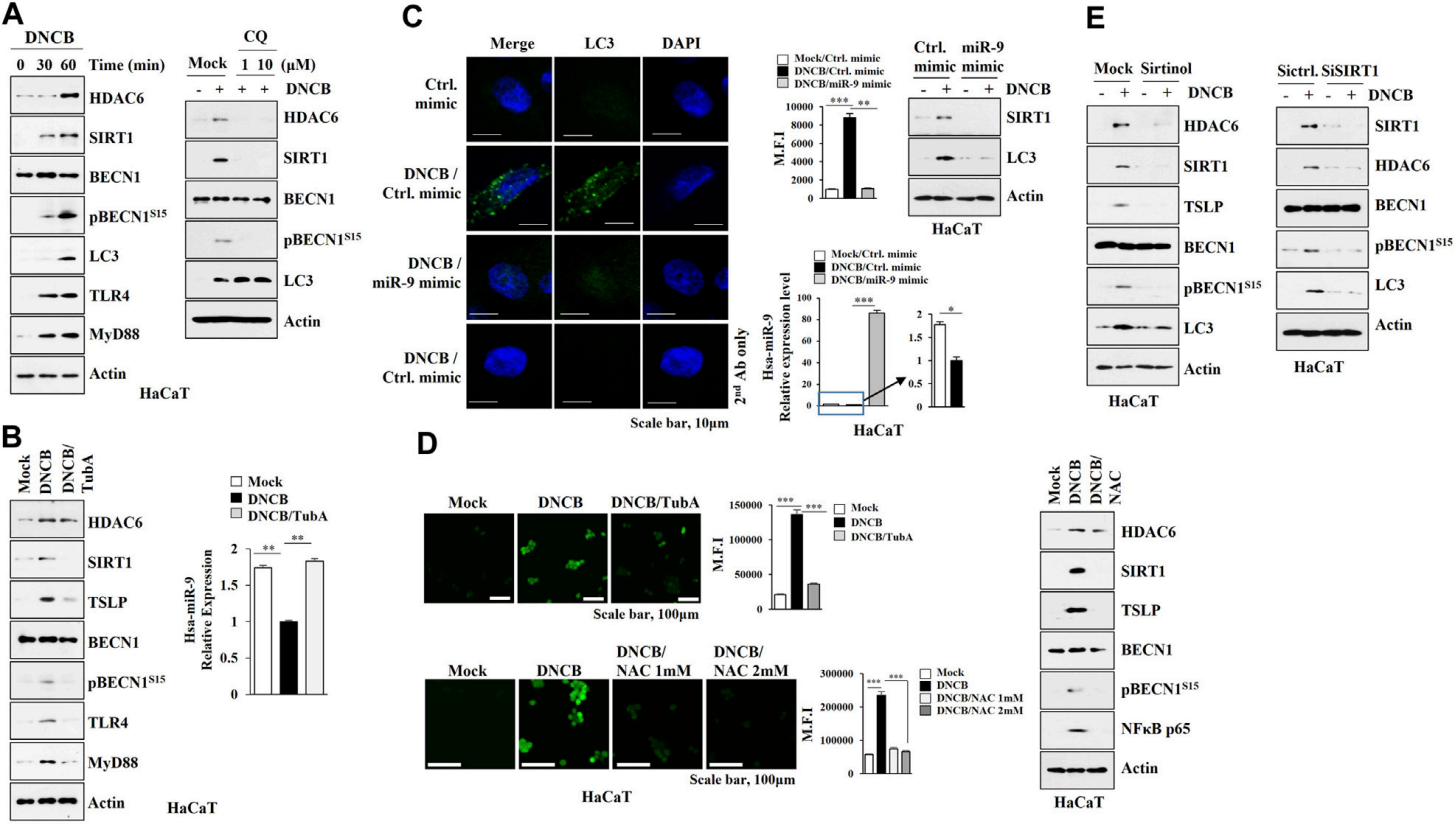
HDAC6-miR-9 axis regulates DNCB-induced autophagic flux and ROS in keratinocytes. **(A)** HaCaT cells were treated with DNCB (5 μM) for various time intervals (left). Cells were pretreated with CQ (10 μM) for 2 h, and treatment with DNCB for 1 h was followed (right). Representative blots of three independent experiments were shown. **(B)** HaCaT cells were pretreated with tubastain A (10 μM) for 2 h, and treatment with DNCB for 1 h was followed. Cell lysates were subjected to immunoblot and qRT-PCR. **, *p* < 0.01. Representative blots of three independent experiments were shown. **(C)** HaCaT cells were transfected with the indicated mimic (each at 10 nM) for 48 h, and treatment with DNCB for 1 h was followed. miR-9 mimic prevents DNCB from increasing the number of LC3 puncta. MFI denotes mean fluorescence intensity. Immunoblot and qRT-PCR were also performed. *, *p* < 0.05; **, *p* < 0.01; ***, *p* < 0.001. Average values of three independent experiments were shown. **(D)** HaCaT cells were pretreated with tubastatin A (10 μM) for 2 h, and treatment with DNCB for 1 h was followed (upper). Cells were pretreated with NAC (5 μM) for 2 h followed by treatment with DNCB for 1 h (lower). ROS level was measured. Cells were pretreated with NAC (5 μM) for 2 h, and treatment with DNCB for 1 h was followed. Immunoblot was then performed (right). ***, *p* < 0.001. **(E)** HaCaT cells were pretreated with sirtinol (10 μM) for 2 h, treatment with DNCB for 1 h was followed (upper). Cells were transfected with the indicated siRNA (each at 10 nM) for 48 h, and treatment with DNCB for 1 h was followed (lower). Immunoblot was performed. Representative blots of three independent experiments were shown.

MicroRNAs that regulate psoriasis and AD are associated with keratinocyte differentiation (Lee et al., 2020) and autophagy in keratinocytes has been reported to be closely related to psoriatic skin inflammation (Wang et al., 2020). A proliferation-inducing ligand (april) from ovalbumin-sensitized mouse skin causes keratinocytes to increase the expression of IL-6 implicated in AD (Leyva-Castillo et al., 2020). These reports led us to hypothesize that keratinocytes may contribute to the pathogenesis of AD. DNCB increased the expression levels of HDAC6, SIRT1, and autophagic flux in HaCaT cells (Figure 7A), while chloroquine (CQ), an autophagy inhibitor, prevented DNCB mediated increases in the expression levels of HDAC6 and SIRT1 (Figure 7A). Tubastatin A prevented DNCB from increasing the expression levels of SIRT1, TSLP, TLR4, Myd88, and pBeclin1^S15^, and decreasing the expression of miR-9 (Figure 7B). The miR-9 mimic prevented DNCB from increasing the expression levels of SIRT1 and LC3 (Figure 7C). Intracellular reactive oxygen species (ROS) enhance autophagic flux (Cho et al., 2020). N-acetyl-L-cystein (NAC), an inhibitor of ROS formation, prevented DNCB from increasing expression levels of SIRT1, TSLP, and pBeclin1^S15^ (Figure 7D). SIRT1 was responsible for the increased expression levels of HDAC6, TSLP, and autophagic flux by DNCB in HaCaT cells (Figure 7E). Thus, enhanced autophagy and ROS signaling by DNCB in keratinocytes may contribute to the development of AD.

### HDAC6 and CXCL13 Mediate Cellular Interactions During AD

**FIGURE 8:**
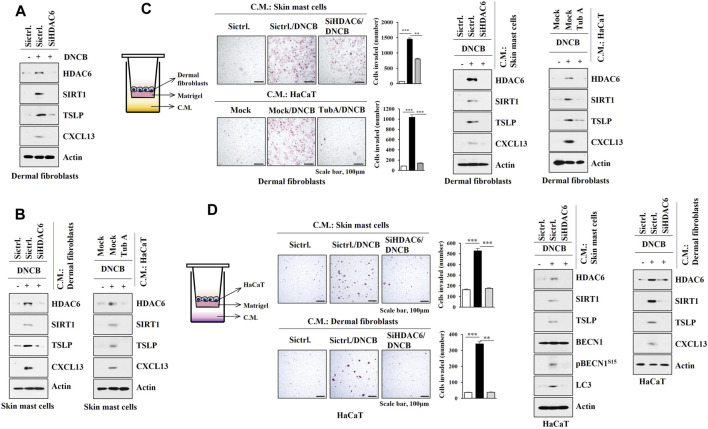
HDAC6 mediates cellular interactions during AD. **(A)** The indicated siRNA (10 nM) was transfected into dermal fibroblast cells. The next day, cells were then treated with DNCB (5 μM) for 1 h followed by immunoblot. Representative blots of three independent experiments were shown. **(B)** Culture medium of dermal fibroblast cells or HaCaT cells was added to skin mast cells for 12 h followed by immunoblot. HaCaT cells were treated with DNCB in the absence or presence of tubastatin A (10 μM) for 2 h. Skin mast cells were isolated from DNCB-untreated Nc/Nga mouse. Representative blots of three independent experiments were shown. **(C)** Transwell assays were performed to determine the effect of culture medium of skin mast cells or HaCaT cell on the invasion of dermal fibroblast cells. Immunoblot of dermal fibroblast cells was also performed. **, *p* < 0.01; ***, *p* < 0.001. Average values of three independent experiments were shown. Skin mast cells were isolated from Nc/Nga mouse of each experimental group and the culture medium was obtained and added to dermal fibroblast cells for 12 h. HaCaT cells were treated as indicated in **(B)**. **(D)** The effect of culture medium of skin mast cells or dermal fibroblast cells on the invasion of HaCaT cells was determined. The lower well contains culture medium. Immunoblot was also performed in HaCaT cells. **, *p* < 0.01; ***, *p* < 0.001. Average values of three independent experiments were shown. Skin mast cells were isolated from Nc/Nga mouse of each experimental group and the culture medium was obtained and added to HaCaT cells for 12 h. Skin dermal fibroblast cells were transfected as indicated. The next day, cells were then treated with DNCB for 1 h.

As AD is associated with the activation of mast cells, keratinocytes, and dermal fibroblast cells, interactions between these cells were examined. DNCB increased the molecular features of AD in dermal fibroblast cells isolated from DNCB-untreated Nc/Nga mice in an HDAC6-dependent manner (Figure 8A). Culture medium of DNCB-treated dermal fibroblast cells and HaCaT cells increased the molecular features of AD in skin mast cells isolated from DNCB-untreated Nc/Nga mouse in an HDAC6-dependent manner (Figure 8B). Culture medium of DNCB-treated skin mast cells and HaCaT cells enhanced invasion of dermal fibroblast cells, and increased the molecular features of AD in dermal fibroblast in an HDAC6-dependent manner (Figure 8C). Culture medium of DNCB-treated skin mast cells and dermal fibroblast cells enhanced the invasion of HaCaT cells and increased the molecular features of AD in HaCaT cells in an HDAC6-dependent manner (Figure 8D). These results suggest that soluble factors may mediate cellular interactions during AD.

**FIGURE 9:**
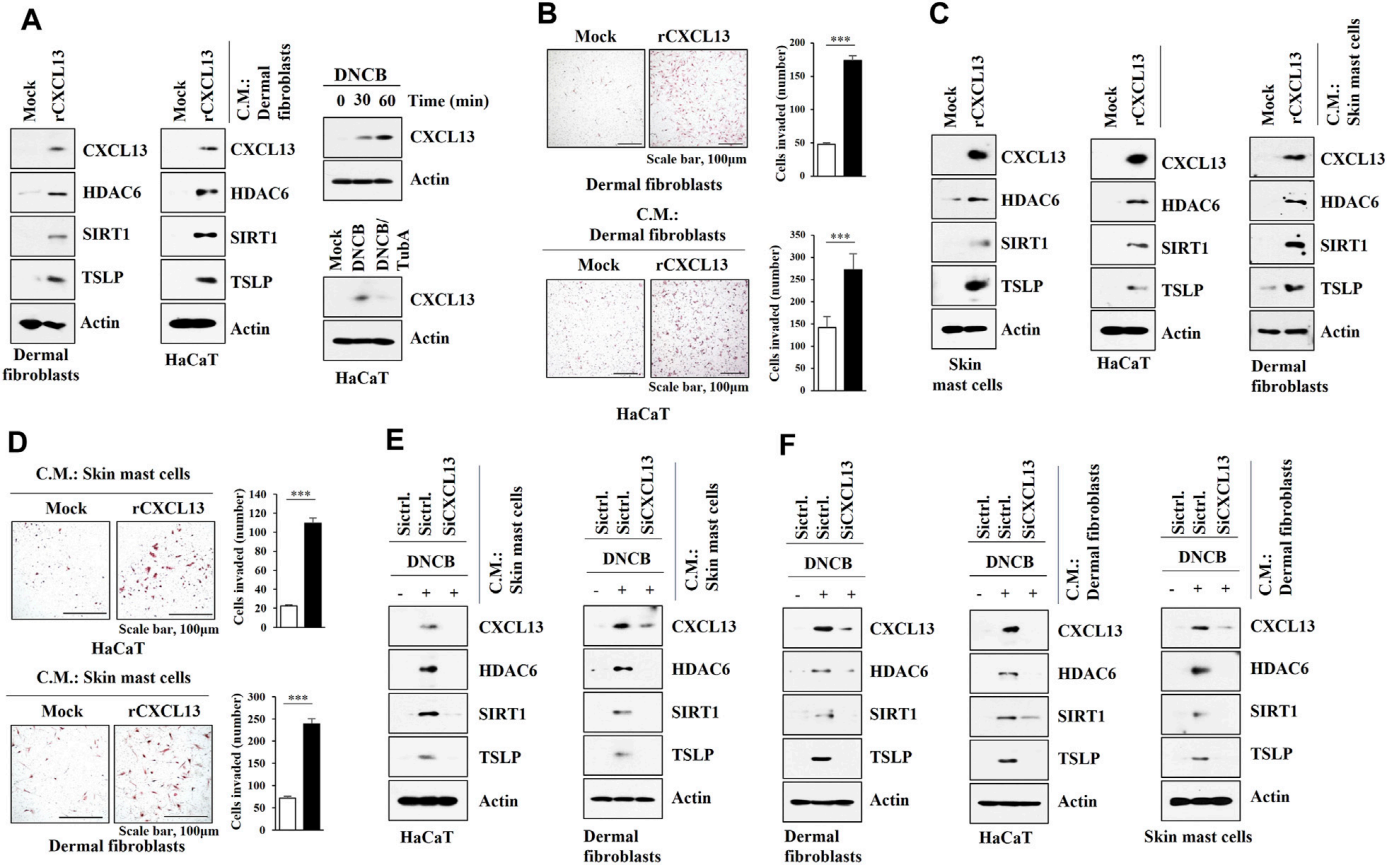
CXCL13 mediates cellular interactions. **(A)** Dermal fibroblast cells were treated with mouse recombinant CXCL13 protein (20 ng) for 2 h (left). Culture medium of dermal fibroblast cells was then added to HaCaT cells for 16 h (middle). HaCaT cells were treated with DNCB (5 μM) for various time intervals (right, upper). HaCaT cells were pretreated with tubastain A (10 μM) for 2 h, and treatment with DNCB (5 μM) for 1 h was followed (right, lower). Immunoblot was performed. Representative blots of three independent experiments were shown. **(B)** Twenty-four hours after treatment with CXCL13 protein, invasion potential of dermal fibroblast cells was determined (upper). Twenty-four hours after treatment with culture medium of skin dermal fibroblast cells treated with recombinant CXCL13 protein for 2 h, invasion potential of HaCaT cells was determined (lower). ***, *p* < 0.001. Average values of three independent experiments were shown. **(C)** Skin mast cells from DNCB-untreated Nc/Nga mouse were treated with mouse recombinant CXCL13 protein (20 ng) for 2 h (left). Culture medium of skin mast cells was then added to HaCaT cells or dermal fibroblast cells for 16 h (right). Immunoblot was performed. Representative blots of three independent experiments were shown. **(D)** Same as **(C)** except that invasion assays were performed as described. Average values of three independent experiments were shown. **(E)** Culture medium of skin mast cells from Nc/Nga mouse of each experimental group was employed. Culture medium of skin mast cells was added to HaCaT cells or dermal fibroblast cells for 16 h. Immunoblot was performed. Representative blots of three independent experiments were shown. **(F)** Indicated siRNA (10 nM) was transfected into dermal fibroblast cells. The next day, cells were then treated with DNCB (5 μM) for 1 h followed by immunoblot (left). Culture medium of dermal fibroblast cells was added to HaCaT cells (middle) or skin mast cells (right) for 16 h followed by immunoblot (right). Representative blots of three independent experiments were shown.

We next examined the possible role of CXCL13 in mediating cellular interactions. Mouse recombinant CXCL13 protein increased the molecular features of AD in dermal fibroblast cells (Figure 9A) and skin mast cells (Figure 9C) and enhanced the invasion of dermal fibroblast cells (Figure 9B). Culture medium of dermal fibroblast cells treated with recombinant CXCL13 protein increased the molecular features of AD in HaCaT cells (Figure 9A) and enhanced the invasion of HaCaT cells (Figure 9B). DNCB also increased CXCL13 expression in an HDAC6-dependent manner in HaCaT cells (Figure 9A). Culture medium of skin mast cells treated with recombinant CXCL13 protein increased the molecular features of AD in HaCaT cells and dermal fibroblast cells (Figure 9C). Culture medium of skin mast cells treated with recombinant CXCL13 protein enhanced the invasion of HaCaT cells and dermal fibroblast cells (Figure 9D). The culture medium of skin mast cells increased the molecular features of AD in HaCaT cells and dermal fibroblast cells in an CXCL13-dependent manner (Figure 9E). The culture medium of DNCB-treated dermal fibroblast cells increased the molecular features of AD in HaCaT cells and skin mast cells in an CXCL13-depenednt manner (Figure 9F).

**FIGURE 10:**
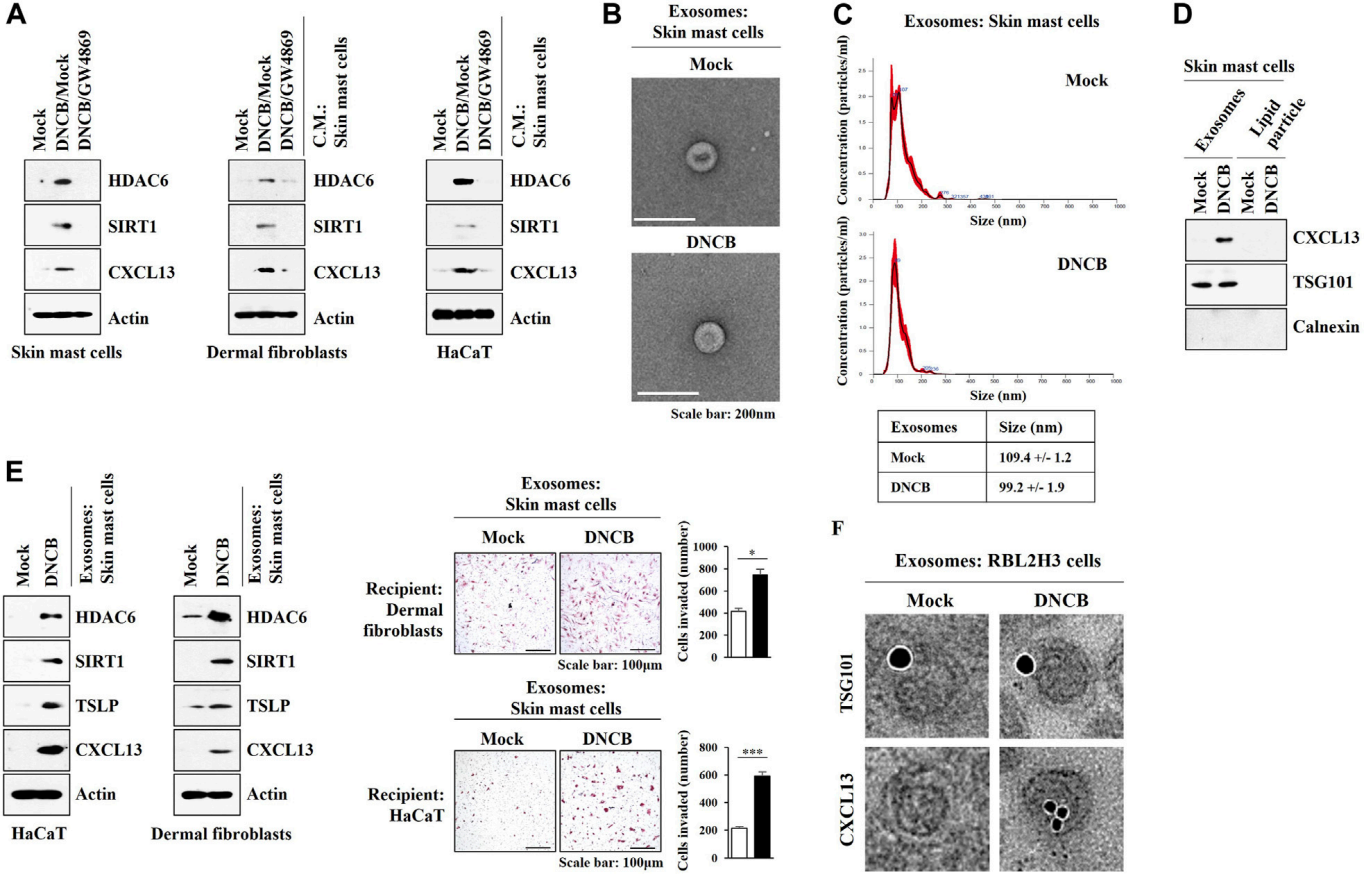
Exosomes of skin mast cells induce features of AD in HaCaT and dermal fibroblast cells. **(A)** Skin mast cells were pretreated with GW4869 (20 μM) for 2 h, and treatment with DNCB (5 μM) for 1 h was followed. HaCaT cells and dermal fibroblast cells were treated with the culture medium of skin mast cells for 12 h. Immunoblot was performed. Representative blots of three independent experiments were shown. **(B)** Exosomes isolated from skin mast cells treated with or without DNCB (5 μM) for 24 h were visualized by negative staining electron microscopy. **(C)** Shows size distributions of exosomes employing nanoparticle tracking analysis (NTA). **(D)** Immunoblot shows the presence of CXCL13 in the exosomes of DNCB-treated skin mast cells. Representative blots of three independent experiments were shown. **(E)** Exosomes were isolated from skin mast cells treated with or without DNCB for 1 h. Exosomes (5 μg) were then added to HaCaT cells or skin dermal fibroblast cells for 24 h followed by immunoblot and invasion assays. *, *p* < 0.05; ***, *p* < 0.001. Average values of three independent experiments were shown. **(F)** Immuno-gold staining images using anti-TSG101, a known membrane marker for the exosomes, and anti-CXCL13 antibody. Twenty-five and 10 nm gold particles show the presence of TSG101 and CXCL13, respectively.

These results led us to hypothesize that exosomes may mediate cellular interactions during AD. GW4869, an inhibitor of exosomes formation, exerted negative effects on the increased expression levels of HDAC6, SIRT1, and CXCL13 by DNCB in skin mast cells isolated from DNCB-untreated Nc/Nga mouse. GW4869 prevented culture medium of skin mast cells treated with DNCB from increasing the molecular features of AD in HaCaT cells and dermal fibroblast cells (Figure 10A). Thus, exosomes may mediate cellular interaction during AD. Exosomes were isolated from DNCB-treated skin mast cells isolated from DNCB-untreated Nc/Nga mouse (Figures 10B,C). The differences in the exosomes sizes measured by TEM (Figure 10B) and nanoparticle tracking analysis (NTA) (Figure 10C) may result from the fact that particles were analyzed in a hydrated and desiccated state, respectively. Immunoblot showed the presence of CXCL13 in the exosomes of skin mast cells (Figure 10D). Purity of the exosomes was confirmed by the absence of calnexin (Figure 10D). Exosomes of DNCB-treated skin mast cells increased the molecular features of AD in HaCaT cells and dermal fibroblast cells and enhanced the invasion of HaCaT cells and dermal fibroblast cells (Figure 10E). Immuno-EM results demonstrated the presence of CXCL13 in exosomes of RBL2H3 cells (Figure 10F). HaCaT cells and skin dermal fibroblast cells showed the uptake of PKH67-labeled exosomes of skin mast cells (Supplementary Figure S3). These results indicate that HDAC6-CXCL13 axis regulates AD by mediating cellular interactions during AD.

## Discussion

Piperlongumine (PPL) suppresses allergic skin inflammation by inhibiting HDAC6 activity (Thatikonda et al., 2020). ACY-1215, an HDAC6 inhibitor, prevents the development of contact hypersensitivity by suppressing the induction of effector T cells (Tsuji et al., 2015). The downregulation (Figure 1) or inhibition (Figure 2) of HDAC6 activity suppressed AD.

The activation of ERK was previously shown to be necessary for AD induced by house dust mite (Dai et al., 2020). DNCB increased the expression of HDAC6 via ERK (Figure 1D). HDAC6 was necessary for the increased phosphorylation of ERK by DNCB (Figure 2D).

IL-37 suppresses AD by increasing the number of Foxp3^+^ regulatory T cells (Treg) and serum level of IL-10 (Hou et al., 2020). Fucoxanthin (FX) inhibits AD by increasing the expression of IL-10 (Natsume et al., 2020). TSA suppresses AD by increasing the number of Treg cells (Kim et al., 2010). AD decreased the expression levels of FoxP3 (Supplementary Figure S1B) and IL-10 in an HDAC6-dependent manner (Supplementary Figure S1C). AD increased expression levels of Th1and Th2 cytokines (Supplementary Figure S1C). It would be interesting to examine the role of T cells in AD by employing T cell ablated mouse model.

DNCB-induced AD was accompanied by an increased formation of autophagosomes (Supplementary Figure S1D). HDAC6 binds to LC3 to mediate autophagy (Peixoto et al., 2020). TLR4/MyD88/ERK signaling mediates Melatonin-induced autophagy (Yang et al., 2020). HDAC6 binds to MyD88 and regulates autophagy (Moreno-Gonzalo et al., 2017). AD induced binding of HDAC6 to MyD88 (Figure 2D), suggesting a role for HDAC6 in autophagy-mediated AD.

CXCL13 was regulated by served as a target of HDAC6 during AD (Figure 3A) and mouse recombinant CXCL13 protein increased the molecular features of AD in skin mast cells (Figure 3C). Necessary to identify downstream targets of CXCL13. It is probable that CXCL13 may regulate the expression levels of Th1/Th2 cytokines during AD.

The HDAC inhibitor Belinostat attenuates skin barrier defects associated with AD by increasing the expression of miR-335 (Liew et al., 2020). MiR-122a inhibits AD by decreasing the expression of SOCS1 (Kim et al., 2018). These reports suggest roles of miRNAs in AD. MicroRNA array results showed miR-9 and miR-222 as targets of HDAC6 (Figure 4A). While the role of miR-222 in AD remains to be seen, The miR-9 mimic suppressed AD (Figure 4B).

MiR-9 inhibits autophagy via targeting Beclin1 (Zhang et al., 2017) and suppresses the proliferation of malignant melanoma cells by targeting SIRT1 (Bu et al., 2017). It is probable that miR-9 mimic may inhibit the effect of DNCB on the formation of autophagosomes. It is known that microRNAs induce epigenetic modifications (Nedoszytko et al., 2020). It would be interesting to examine the effect of miR-9 on the methylation status of genes involved in AD.

We identified SIRT1 as a direct target of miR-9 (Figure 5A). SIRT1 is necessary for mast cell signaling and anaphylaxis (Li et al., 2018), and mediates allergic airway inflammation via autophagy (Wu et al., 2020). SIRT1/NF-kB/TLR2 signaling promotes inflammatory responses in macrophages (Qian et al., 2018). SIRT1was shown to mediate AD (Figure 6A). Thus, SIRT1/TLR signaling likely plays a role in AD by mediating cellular interactions involving mast cells and macrophages.

Keratinocyte-derived cytokines, such as TSLP, contributes to the pathogenesis of AD (Sawada et al., 2019). while Keratinocytes from patients with AD show increased level of PGE2 (Seo et al., 2020). DNCB increased the expression level of TSLP in HaCaT cells in an HDAC6-dependent manner (Figure 7B).

Allergic skin inflammation is closely related with the increased reactive oxygen species (ROS) level (Keum et al., 2020) and AD keratinocytes show an increased ROS level (Emmert et al., 2020). ROS/MAPK signaling activates autophagic flux (Shen et al., 2020). Increased ROS by hypoxia enhances migration of keratinocytes and promotes autophagy (Zhang L. et al., 2019). HDAC6 promotes inflammation by enhancing ROS signaling (Youn et al., 2016; Zhang WB. et al, 2019), and HDAC6 was necessary for the increased ROS levels caused by DNCB in HaCaT cells (Figure 7D). NAC prevented DNCB from increasing the expression level of pBeclin1^S15^ in HaCaT cells (Figure 7D). Thus, NAC may suppress AD by inhibiting autophagic flux.

The interaction between eosinophils and dermal fibroblast cells contributes to the pathogenesis of AD (Hou et al., 2020). The deletion of IKK2 in dermal fibroblast cells leads to skin inflammation (Nunomura et al., 2019). Atopic fibroblast cells regulate differentiation of keratinocytes during AD pathogenesis (Berroth et al., 2013). TWEAK mediates AD by upregulating TSLP in keratinocytes and dermal fibroblast cells (Sidler et al., 2017). Thus, cellular interactions involving keratinocytes and fibroblast cells may play roles in AD.

Experiments employing culture medium and transwell showed that DNCB-activated skin mast cells induced molecular features of AD in dermal fibroblast cells (Figure 8C) and keratinocytes (Figure 8D) in an HDAC6-dependent manner. We hypothesized that soluble factors could mediate these cellular interactions. Mouse recombinant CXCL13 protein also increased the molecular features of AD in dermal fibroblast cells (Figure 9A), suggesting that CXCL13 may mediate cellular interactions involving mast cells and dermal fibroblast cells during AD. Experiments employing culture medium and transwell showed that CXCL13 mediated cellular interactions during AD.

Roles of Langerhans cells, and inflammatory dendritic epidermal cells (IDEC) in AD have been reported (Klaeschen et al., 2021; Marschall et al., 2021). It would be necessary to examine interactions between mast cells, Langerhans cells, and IDECs by employing culture medium and transwell assays.

Pathogen-derived exosomes induce AD-like skin inflammation by upregulating inflammatory cytokines in dermal fibroblast cells (Hong et al., 2011). Exosomes from adipose tissue-derived stem cells inhibit molecular features of AD such as M2 macrophages polarization (Kim et al., 2018). Exosomes from adipose tissue-derived stem cells attenuates clinical symptoms associated with AD by promoting lipid synthesis (Shin et al., 2020). Thus, exosomes may regulate AD by mediating cellular interactions. We showed that exosomes from DNCB-activated mast cells induced features of AD in dermal fibroblast cells and keratinocytes (Figure 10E). Exosomal cytokines and miRNAs would regulate AD by mediating cellular interactions.

AD is related with the increased production of TH17-associated cytokines (Noda et al., 2015). AD lesion shows upregulation of TH17 activity (Wongvibulsin et al., 2021). It would be necessary to examine the effect of HDAC6 and CXCL13 on TH17 activity.

In conclusion, HDAC6-CXCL13 regulates AD by mediating cellular interactions and can be employed as target for the development of anti-atopic drugs.

**TABLE 1:** Primer sequences used for qRT-PCR.

Name	Forward primer	Reverse primer
HDAC6	CCT​CAG​CGC​ATC​TTA​CGC​AT	CAG​CAC​TGT​GGC​AGG​TAA​GG
IL-4	AAC​GAG​GTC​ACA​GGA​GAA​GG	TCT​GCA​GCT​CCA​TGA​GAA​CA
IL-5	GGC​CAC​TGC​CAT​GGA​GAT​TC	GGA​AGC​CTC​ATC​GTC​TCA​TTG​C
IL-13	GCA​GCA​TGG​TAT​GGA​GTG​TG	TGG​CGA​AAC​AGT​TGC​TTT​GT
IL-1β	ATG​CCA​CCT​TTT​GAC​AGT​GAT​G	TGT​GCT​GCT​GCG​AGA​TTT​GA
IFNγ	TCA​AGT​GGC​ATA​GAT​GTG​GAA​GAA	TGG​CTC​TGC​AGG​ATT​TTC​ATG
TNF-α	CCC​AAA​TGG​CCT​CCC​TCT​C	GTT​TGC​TAC​GAC​GTG​GGC​TA
IL-10	GGC​GCT​GTC​ATC​GAT​TTC​TCC	TGG​CCT​TGT​AGA​CAC​CTT​GGT​C
CXCL13	ATG​TGT​GAA​TCC​TCG​TGC​CA	CAC​TGG​AGC​TTG​GGG​AGT​TG
SIRT1	TTG​TGA​AGC​TGT​TCG​TGG​AG	GGC​GTG​GAG​GTT​TTT​CAG​TA
Actin	ATG​TGG​ATC​AGC​AAG​CAG​GA	CTA​GAA​GCA​CTT​GCG​GTG​C
MiR-9	TCT​TTG​GTT​ATC​TAG​CTG​TAT​GA	—
U6	TGG​CCC​CTG​CGC​AAG​GAT​G	—

**TABLE 2:** Primary antibodies used for immunoblot and immunohistochemical staining.

Catalog No	Name	Catalog No	Name
A11259	HDAC6	ab215206	FoxP3
A11225	TLR2	ab188766	TSLP
A5258	TLR4	ab9950	MIP-2
sc-393789	FcεRIβ	ab115819	iNOS
sc-7274	Lyn	#13120	iNOS
sc-268	GATA3	#84966	pBECN1^S14^
sc-21749	T-bet	#12282	COX2
sc-1648	JNK1	#4695	ERK1/2
sc-6254	pJNK1^T183/Y185^	#4370	pERK^T204^
sc-59587	Tryptase	#3949	HDAC3
sc-59586	Chymase	#8242	NFκB
sc-48341	BECN1	#5831	AMPKα
sc-74532	MyD88	#50081	pAMPKα^T172^
sc-7964	TSG101	#4812	IKBα
sc-23954	Calnexin	#2859	pIKBα^S32^
sc-74465	SIRT1	#8690	p38MAPK
13161-1-AP	SIRT1	#4511	p-p38MAPK^T180/Y182^
AF470	CXCL13	#4108	LC3
ab182422	CD163	—	—

## Data Availability

The original contributions presented in the study are included in the article/Supplementary Material, further inquiries can be directed to the corresponding author.

## Abbreviations

AD, atopic dermatitis; COX2, cyclooxygenase 2; CQ, chloroquine; CXCL13, CXC chemokine ligand 13; DNCB, 2,4- dinitrochlorobenzene; HDAC6, histone deacetylase 6; MAPK, mitogen activated protein kinase; MiRNA, microRNA; PGE2, prostaglandin E2; ROS, reactive oxygen species; SiRNA, small interfering RNA; SIRT1, NAD-dependent deacetylase sirtuin-1; TSLP, thymic stromal lymphopoietin; TWEAK, TNF-related weak inducer of apoptosis.
