# Supplementary material for: HDAC6 and CXCL13 Mediate Atopic Dermatitis by Regulating Cellular Interactions and Expression Levels of miR-9 and SIRT1
Source: Front Pharmacol. 2021 Sep 13;12:691279. doi: 10.3389/fphar.2021.691279 (PMC8473914; doi:10.3389/fphar.2021.691279)
Supplement: Supplementary file 1 [file DataSheet1.docx]

Supplementary Material

**Table1.** Primer sequences used for qRT-PCR

| Name | Forward primer | Reverse primer |
| --- | --- | --- |
| HDAC6 | CCTCAGCGCATCTTACGCAT | CAGCACTGTGGCAGGTAAGG |
| IL-4 | AACGAGGTCACAGGAGAAGG | TCTGCAGCTCCATGAGAACA |
| IL-5 | GGCCACTGCCATGGAGATTC | GGAAGCCTCATCGTCTCATTGC |
| IL-13 | GCAGCATGGTATGGAGTGTG | TGGCGAAACAGTTGCTTTGT |
| IL-1β | ATGCCACCTTTTGACAGTGATG | TGTGCTGCTGCGAGATTTGA |
| IFNγ | TCAAGTGGCATAGATGTGGAAGAA | TGGCTCTGCAGGATTTTCATG |
| TNF-α | CCCAAATGGCCTCCCTCTC | GTTTGCTACGACGTGGGCTA |
| IL-10 | GGCGCTGTCATCGATTTCTCC | TGGCCTTGTAGACACCTTGGTC |
| CXCL13 | ATGTGTGAATCCTCGTGCCA | CACTGGAGCTTGGGGAGTTG |
| SIRT1 | TTGTGAAGCTGTTCGTGGAG | GGCGTGGAGGTTTTTCAGTA |
| Actin | ATGTGGATCAGCAAGCAGGA | CTAGAAGCACTTGCGGTGC |
| MiR-9 | TCTTTGGTTATCTAGCTGTATGA | - |
| U6 | TGGCCCCTGCGCAAGGATG | - |

**Table2.** Primary antibodies used for immunoblot and immunohistochemical staining

| Catalog No. | Name | Catalog No. | Name |
| --- | --- | --- | --- |
| A11259 | HDAC6 | ab215206 | FoxP3 |
| A11225 | TLR2 | ab188766 | TSLP |
| A5258 | TLR4 | ab9950 | MIP-2 |
| sc-393789 | FcεRIβ | ab115819 | iNOS |
| sc-7274 | Lyn | #13120 | iNOS |
| sc-268 | GATA3 | #84966 | pBECN1^S14^ |
| sc-21749 | T-bet | #12282 | COX2 |
| sc-1648 | JNK1 | #4695 | ERK1/2 |
| sc-6254 | pJNK1^T183/Y185^ | #4370 | pERK^T204^ |
| sc-59587 | Tryptase | #3949 | HDAC3 |
| sc-59586 | Chymase | #8242 | NFκB |
| sc-48341 | BECN1 | #5831 | AMPKα |
| sc-74532 | MyD88 | #50081 | pAMPKα^T172^ |
| sc-7964 | TSG101 | #4812 | IKBα |
| sc-23954 | Calnexin | #2859 | pIKBα^S32^ |
| sc-74465 | SIRT1 | #8690 | p38MAPK |
| 13161-1-AP | SIRT1 | #4511 | p-p38MAPK^T180/Y182^ |
| AF470 | CXCL13 | #4108 | LC3 |
| ab182422 | CD163 |  |  |

**Supplemantary figures**


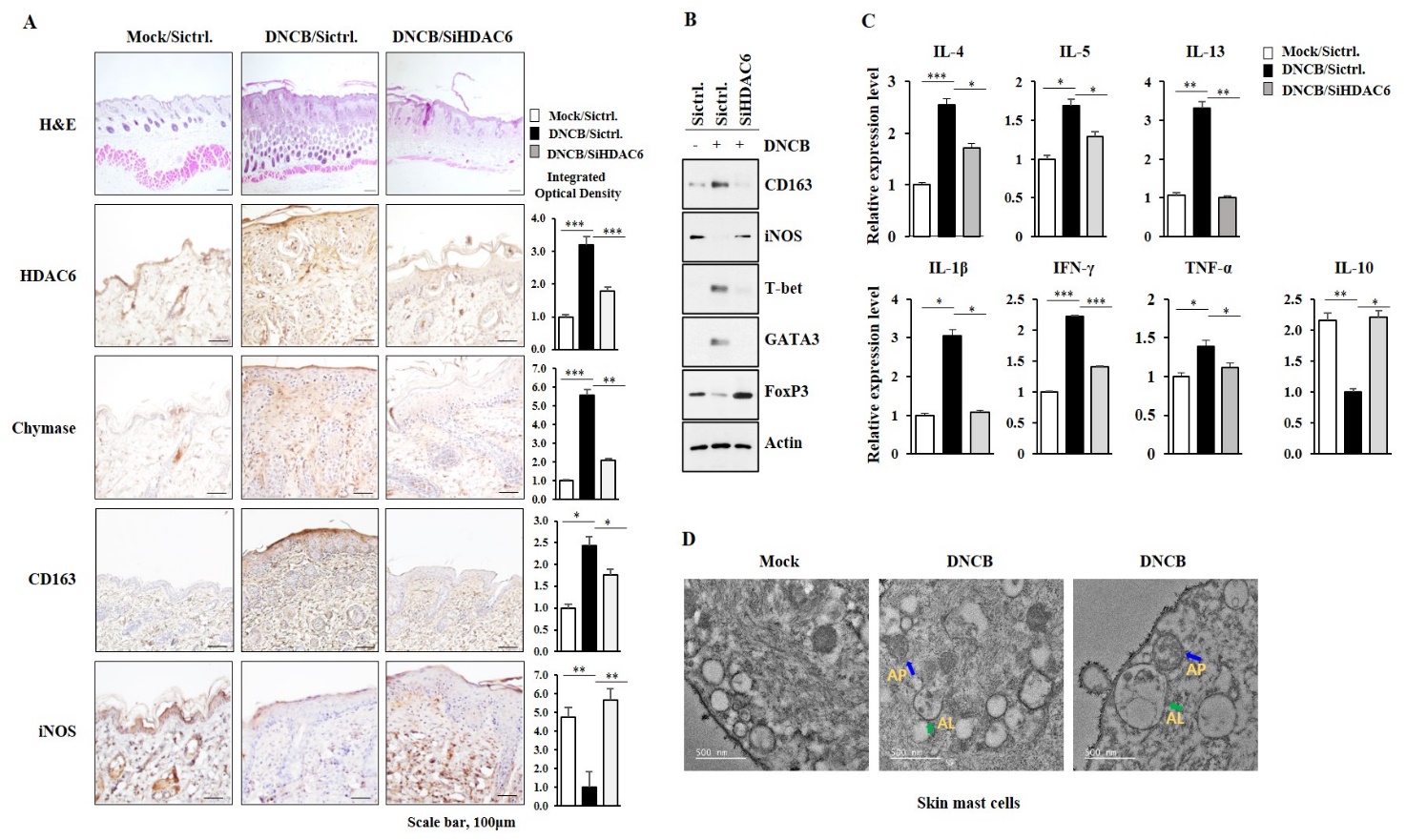


**Figure S1.** HDAC6 regulates molecular and cellular features of AD. **(A)** Immunohistochemical staining and H&E staining were performed. *, *p*<0.05; **, *p*<0.01; ***, *p*<0.001. Quantiﬁcation was performed by calculating the percentage of the staining intensities using Image J (NIH). **(B)** Skin tissue lysates were subjected to immunoblot. Representative blots of three independent experiments were shown. **(C)** QRT-PCR employing skin tissue lysates was performed. *, *p*<0.05; **, *p*<0.01; ***, *p*<0.001. Average values of three independent experiments were shown. **(D)** DNCB enhances autophagic processes. Representative electron micrographs of skin mast cells treated without or with DNCB (5 μM) for 2 h were shown. AL: autolysosomes; AP: autophagosomes. Skin mast cells were isolated from DNCB-untreated Nc/Nga mouse.


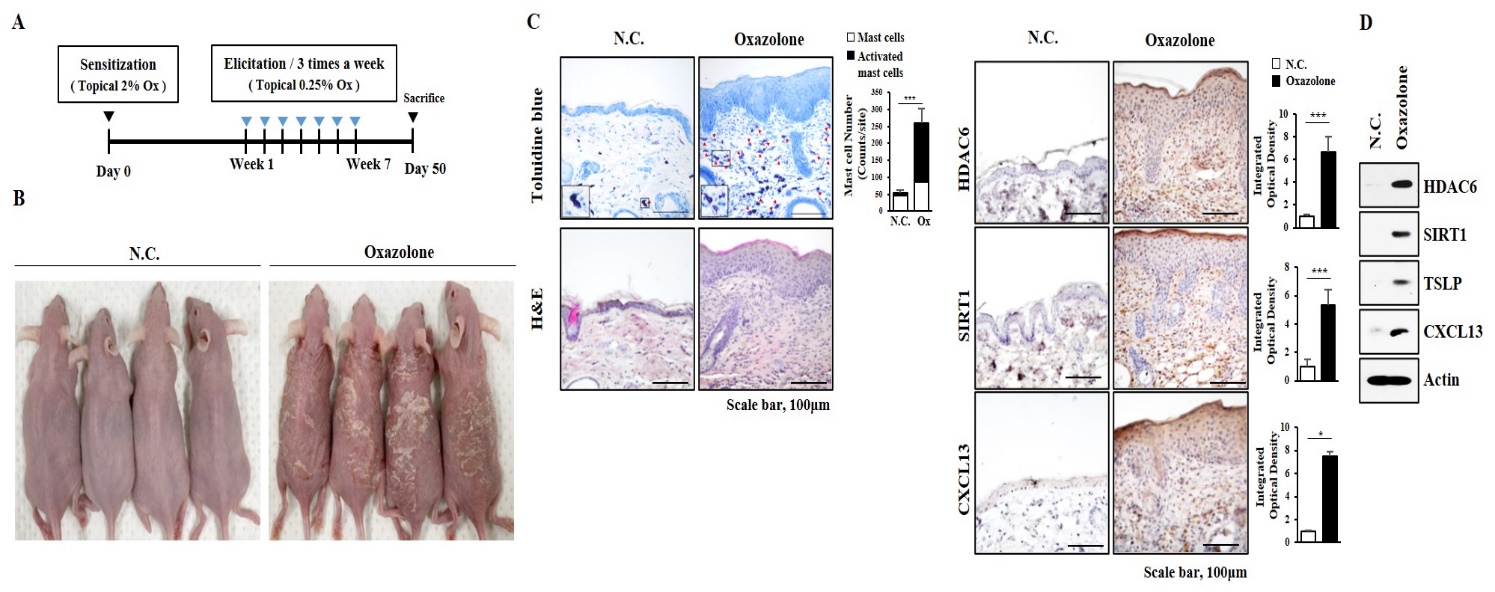


**Figure S2.** Oxazolone-induced AD is accompanied by the increased expression levels of HDAC6 and SIRT1. **(A)** Shows schemes of the experimental procedures. **(B)** Shows AD-like skin lesions induced by oxazolone. **(C)** H&E staining, toluidine blue staining, and immunohistochemical staining were performed. Closed triangle represents degranulated mast cells. *, *p*<0.05; ***, *p*<0.001. Quantiﬁcation was performed using Image J (NIH). **(D)** Immunoblot of skin tissue lysates was performed. Representative blots of three independent experiments were shown.


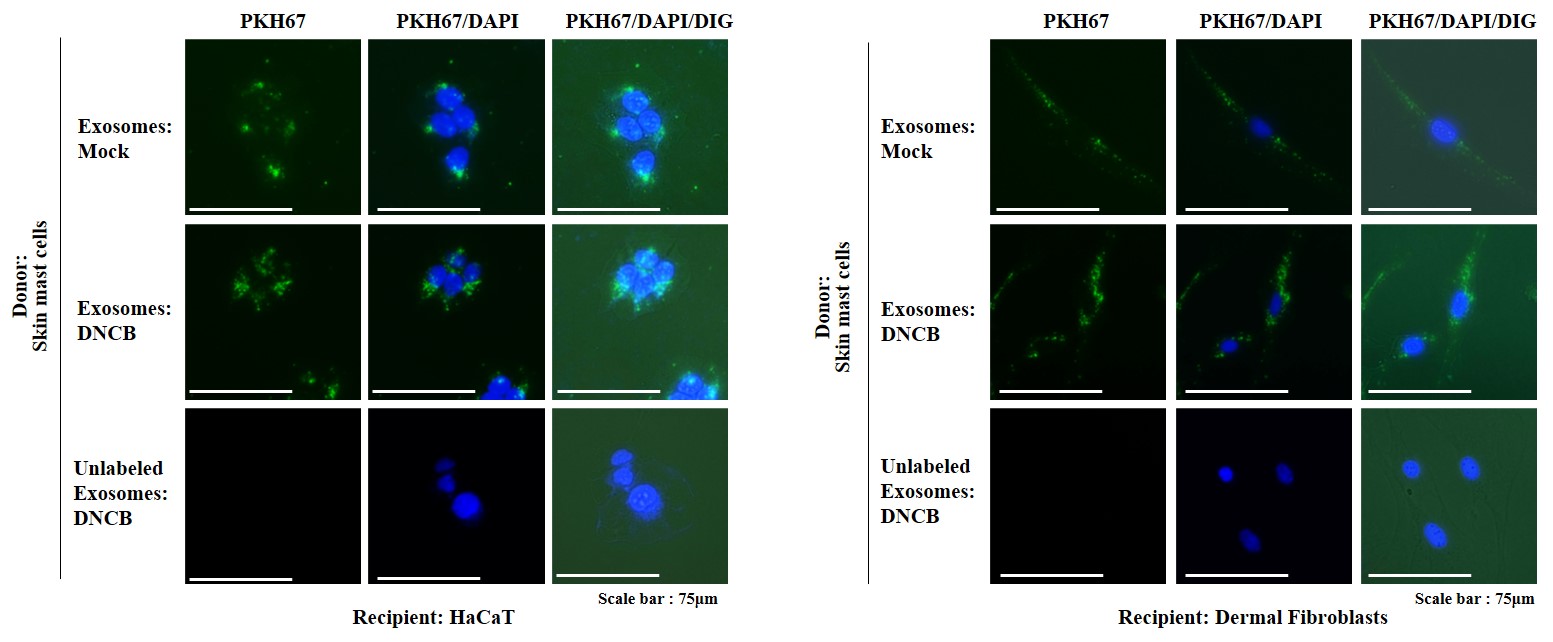


**Figure S3.** Exosomes shuttle between cells. Exosomes were isolated from skin mast cells treated without or with DNCB (5 μM) for 24 h. PKH67-labeled exosomes (2 μg) or unlabeled exosomes (2 μg) were added to HaCaT or skin dermal fibroblast cells for 24 h. Cells were then visualized using EVOS FL Auto 2 (ThermoFisher, USA).
